# Loss of LKB1-NUAK1 signalling enhances NF-κB activity in a spheroid model of high-grade serous ovarian cancer

**DOI:** 10.1038/s41598-022-06796-2

**Published:** 2022-02-22

**Authors:** Adrian Buensuceso, Jamie Lee Fritz, Olga Collins, Yudith Ramos Valdés, Matthew J. Borrelli, Gabriel E. DiMattia, Trevor G. Shepherd

**Affiliations:** 1 The Mary & John Knight Translational Ovarian Cancer Research Unit, London Regional Cancer Program, 790 Commissioners Road East, Room A4-836, London, ON N6A 4L6 Canada; 2 Department of Anatomy and Cell Biology, Schulich School of Medicine and Dentistry, Western University, London, ON Canada; 3 Department of Biochemistry, Schulich School of Medicine and Dentistry, Western University, London, ON Canada; 4 Department of Oncology, Schulich School of Medicine and Dentistry, Western University, London, ON Canada; 5 Department of Obstetrics and Gynaecology, Schulich School of Medicine and Dentistry, Western University, London, ON Canada

**Keywords:** Cancer, Cell biology

## Abstract

High-grade serous ovarian cancer (HGSOC) is an aggressive malignancy often diagnosed at an advanced stage. Although most HGSOC patients respond initially to debulking surgery combined with cytotoxic chemotherapy, many ultimately relapse with platinum-resistant disease. Thus, improving outcomes requires new ways of limiting metastasis and eradicating residual disease. We identified previously that Liver kinase B1 (LKB1) and its substrate NUAK1 are implicated in EOC spheroid cell viability and are required for efficient metastasis in orthotopic mouse models. Here, we sought to identify additional signalling pathways altered in EOC cells due to LKB1 or NUAK1 loss-of-function. Transcriptome analysis revealed that inflammatory signalling mediated by NF-κB transcription factors is hyperactive due to LKB1-NUAK1 loss in HGSOC cells and spheroids. Upregulated NF-κB signalling due to NUAK1 loss suppresses reactive oxygen species (ROS) production and sustains cell survival in spheroids. NF-κB signalling is also activated in HGSOC precursor fallopian tube secretory epithelial cell spheroids, and is further enhanced by NUAK1 loss. Finally, immunohistochemical analysis of OVCAR8 xenograft tumors lacking NUAK1 displayed increased RelB expression and nuclear staining. Our results support the idea that NUAK1 and NF-κB signalling pathways together regulate ROS and inflammatory signalling, supporting cell survival during each step of HGSOC pathogenesis. We propose that their combined inhibition may be efficacious as a novel therapeutic strategy for advanced HGSOC.

## Introduction

Epithelial ovarian cancer (EOC) is the most lethal gynaecologic malignancy in the developed world and is typically diagnosed after it has already metastasized. Consequently, fewer than 30% of patients survive longer than five years after diagnosis^1^. While the majority of advanced-stage EOC patients initially respond to cytoreductive surgery and combination chemotherapy, most will ultimately relapse with chemo-resistant disease^2^. Therefore, increased understanding of the molecular and cellular adaptations supporting the survival and growth of EOC cells during metastasis may yield novel approaches to improve patient outcomes.

Liver kinase B1 (LKB1; encoded by *STK11* in humans) is a serine-threonine kinase that is expressed in most cell types and is recognized as a master regulator of metabolism^3^. LKB1 and its direct target substrate AMP-activated protein kinase (AMPK) comprise an integral signalling axis that senses depletion of intracellular ATP levels, enabling restoration of energy homeostasis and permitting survival during metabolic stress^4^. While LKB1 has tumour-suppressive function in several disease contexts^5–11^, a growing body of evidence supports the notion that it can also promote cancer metastasis^12–15^. Although AMPK is the most well-characterized substrate of LKB1, our recent data indicate that LKB1 acts independently of AMPK to elicit pro-survival mechanisms in EOC metastasis^16^. There are several other known LKB1 substrates^17^, such as the AMPK-related kinases (ARKs), which consist of brain-specific kinases (BRSKs), involved in promoting neuronal polarity^18^, salt-inducible kinases (SIKs), involved in regulating cell polarity, proliferation, apoptosis, and lipid metabolism^19^, the nua kinases (NUAKs), which regulate adhesion^20^, motility^21^, proliferation^22^ and stress responses^23^, the microtubule affinity-regulating kinases (MARKs), which regulate cell polarity and motility^24^, and sucrose non-fermenting related kinase (SNRK), which is expressed primarily in testes and may regulate spermatogenesis^25^. Through a proteomic screen followed by direct functional analysis, we determined that LKB1 regulates NUAK1 expression and phosphorylation in EOC cells, and that LKB1-NUAK1 activity is required for metastasis using in vitro and in vivo models of the disease^16,20^, consistent with a previous report linking NUAK1 with poor prognosis in ovarian cancer^26^.

LKB1 has been implicated in inflammatory pathway regulation in multiple contexts^27,28^, including cancer^29^. Inflammation evolved as a protective response to trauma, becoming activated when an organism is subjected to injury, toxicity, or infection^30^. Commonly, this response acts to recruit immune cells to the site of damage to neutralize pathogens while simultaneously regulating tissue repair pathways^31^. Nuclear factor kappa-light-chain-enhancer of activated B cells (NF-κB) signalling is well-established as a key proinflammatory regulator mediated by two different pathways^32^. The canonical pathway has perhaps been best characterized in the context of inflammation and is typically activated by numerous proinflammatory cytokines, which act through cell surface receptors to activate the IκB kinase complex via transforming growth factor beta-activated kinase 1 (TAK1)^33,34^ and its associated proteins, leading to nuclear translocation of NF-κB transcription factors and target gene regulation. In contrast, non-canonical NF-κB pathway activation is mediated by NF-κB-inducing kinase (NIK)^35^, which regulates the proteolytic processing of NF-κB subunit p100 into its active p52 form^36,37^, leading to dimerization with RelB to affect NF-κB target gene expression. In addition to activation by proinflammatory cytokines, NF-κB signalling may become activated in response to various cellular stresses, such as oxidative stress^38^, ionizing radiation^39^, and ultraviolet light^40^.

LKB1 activity has been demonstrated as having a repressive effect on NF-κB signalling^27,28,41^, while the role of NUAK1 in regulating NF-κB proinflammatory signalling is unknown. However, NUAK1 is required in colorectal cancer cells for oxidative stress-induced nuclear factor erythroid 2-related factor (NRF2) activation^23^, which can suppress proinflammatory cytokine gene expression^42^. As such, it is plausible that NUAK1 has context-specific effects on inflammatory signalling. Herein, we show that genetic ablation of LKB1 or NUAK1 in EOC cells is associated with overactive NF-κB signalling, and is further exacerbated in multicellular clusters, or spheroids. However, we demonstrate that there are differences between LKB1 and NUAK1 in utilization of NF-κB signalling to elicit gene expression changes. Notably, elevated NF-κB transcription factor nuclear localization and inflammatory gene expression is correlated with decreased reactive oxygen species (ROS) levels and increased cell viability in spheroids lacking NUAK1, effects that are negated by treatment with the IkB kinase (IKK) inhibitor BAY 11-7082.

## Results

### Inflammation-associated transcriptional signatures in EOC cells lacking LKB1 or NUAK1

**Figure 1:**
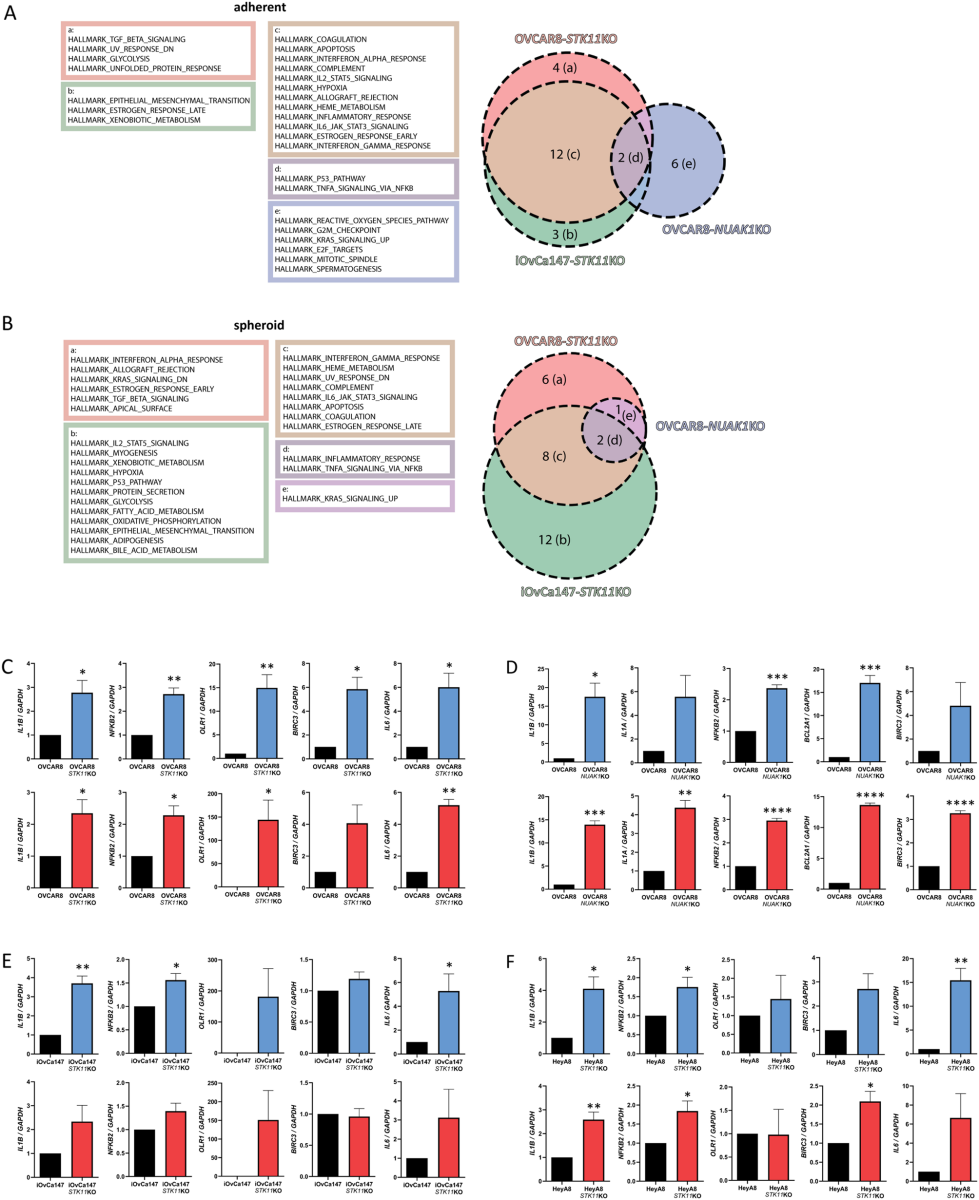
Enrichment of inflammation-associated transcriptional signatures in EOC cell lines lacking LKB1 or NUAK1. (**A**, **B**) Transcriptional signatures enriched in *STK11*KO or *NUAK1*KO cells as compared with parental EOC cells in adherent culture (**A**) or spheroid culture (**B**). Lists of Hallmarks were determined using Gene Set Enrichment Analysis (BROAD Institute; p-value cutoff: 0.05, FDR cutoff: 0.25). (**C**–**F**) Expression of selected NF-κB-target genes by RT-qPCR in OVCAR8-*STK11*KO (**C**), OVCAR8-*NUAK1*KO (**D**), iOvCa147-*STK11*KO (**E**), and HeyA8-*STK11*KO (**F**) cell lines. Data from adherent culture of knockout cell lines shown in blue and spheroid culture of knockout cell lines are shown in red. Knockout cells were compared with parental cell controls by unpaired, two-tailed Student’s *t*-test (*p ≤ 0.05, **p ≤ 0.01, ***p ≤ 0.001, ****p ≤ 0.0001; n = 3). Error bars indicate standard error of the mean (S.E.M.).

We previously reported that knockout of LKB1 in EOC cells results in decreased metastatic potential both in vitro and in vivo^16,43^, while NUAK1 ablation was more limited to altered cell adhesion and reduced tumour growth^20^. To interrogate additional molecular mechanisms underlying these phenotypes due to LKB1 and NUAK1 loss, we performed microarray-based transcriptome and subsequent pathway analysis. Gene set enrichment analysis^44,45^ revealed enrichment of the HALLMARK_TNFA_SIGNALING_VIA_NFKB signature in EOC cells lacking LKB1 or NUAK1 as compared with control cells in both adherent and spheroid culture conditions (Fig. 1A, B). Interestingly, this gene set was also enriched in spheroids as compared with adherent cells for both OVCAR8 and iOvCa147 cell lines (Supplementary Fig. S2A). We validated our microarray results by RT-qPCR in which specific NF-κB target genes were significantly increased in OVCAR8-*STK11*KO or OVCAR8-*NUAK1*KO cells and spheroids (Fig. 1C, D). Expression of these genes was assessed in iOvCa147-*STK11*KO and HeyA8-*STK11*KO cells, but their upregulation was observed to a lesser degree for some targets (Fig. 1E, F), consistent with the NF-κB pathway’s ability to selectively activate subsets of target genes in different contexts^46^. Thus, these results indicate that NF-κB transcriptional activity may be enhanced due to the ablation of either LKB1 or NUAK1 activity in EOC cells, particularly in the context of spheroids.

### NF-κB signalling is activated due to LKB1-NUAK1 loss in EOC cells

**Figure 2:**
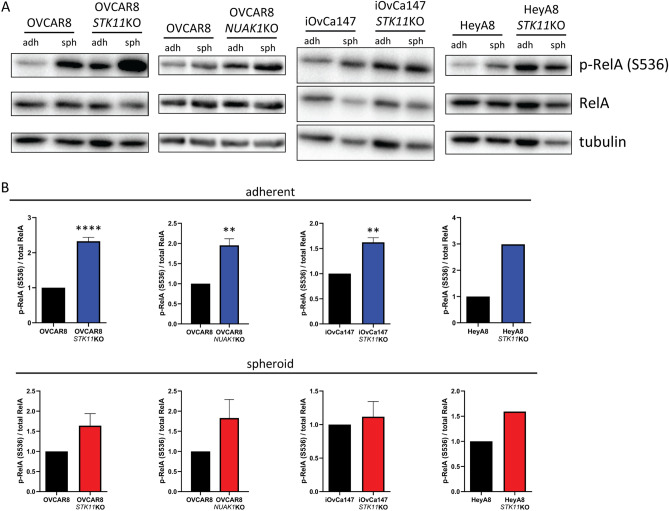
Increased phosphorylation of RelA at S536 in EOC cell lines lacking LKB1 or NUAK1. (**A**) Representative immunoblots for p-RelA-S536 in OVCAR8-*STK11*KO, OVCAR8-*NUAK1*KO, iOvCa147-*STK11*KO, and HeyA8-*STK11*KO cell lines and parental lines grown in adherent (adh) and spheroid (sph) culture conditions. Total RelA and tubulin were used as controls. (**B**) Densitometric analysis of p-RelA-S536 expression with data from adherent culture of knockout cell lines shown in blue and spheroid culture of knockout cell lines are shown in red. Knockout cell lines were compared with parental cell controls (OVCAR8 and iOvCa147) by unpaired, two-tailed Student’s *t*-test (*p ≤ 0.05, **p ≤ 0.01, ****p ≤ 0.001; n = 3). Error bars indicate standard error of the mean (S.E.M.).

Given our transcript-level data indicating enhanced NF-κB target gene expression in EOC cells lacking LKB1 or NUAK1, we directly evaluated NF-κB pathway activation. Canonical NF-κB signalling regulates gene expression primarily via heterodimers of RelA (also referred to as p65) and p50. Transcriptional activity of NF-κB complexes is controlled by a combination of IκB-regulated nuclear localization and various post-translational modifications^47^. For example, phosphorylation of RelA at S536 is associated with its increased transcriptional activity^47,48^. Phospho-RelA (S536) is significantly increased in parental EOC cell line spheroids (Fig. 2A and Supplementary Fig. S2E), consistent with our results indicating an enhanced NF-κB transcriptional signature in spheroids (Supplementary Fig. S2A–D). Under adherent conditions, OVCAR8-*STK11*KO, OVCAR8-*NUAK1*KO, and iOvCa147-*STK11*KO cells exhibit significantly increased p-RelA (S536) compared to parental controls (Fig. 2B). In contrast, no significant differences were observed in *STK11*KO or *NUAK1*KO spheroid cells compared to parental controls (Fig. 2B).

These results indicate that knockout of *STK11* or *NUAK1* is sufficient to increase phosphorylation of RelA at serine 536 under adherent culture conditions, which may explain the enhanced expression of NF-κB target genes in adherent cells due to LKB1/NUAK1 loss. Next, we sought to assess nuclear abundance of several NF-κB family transcription factors: RelA and c-Rel, which mediate gene expression via the canonical NF-κB pathway, and RelB and p52, which mediate gene expression via the non-canonical pathway.

**Figure 3:**
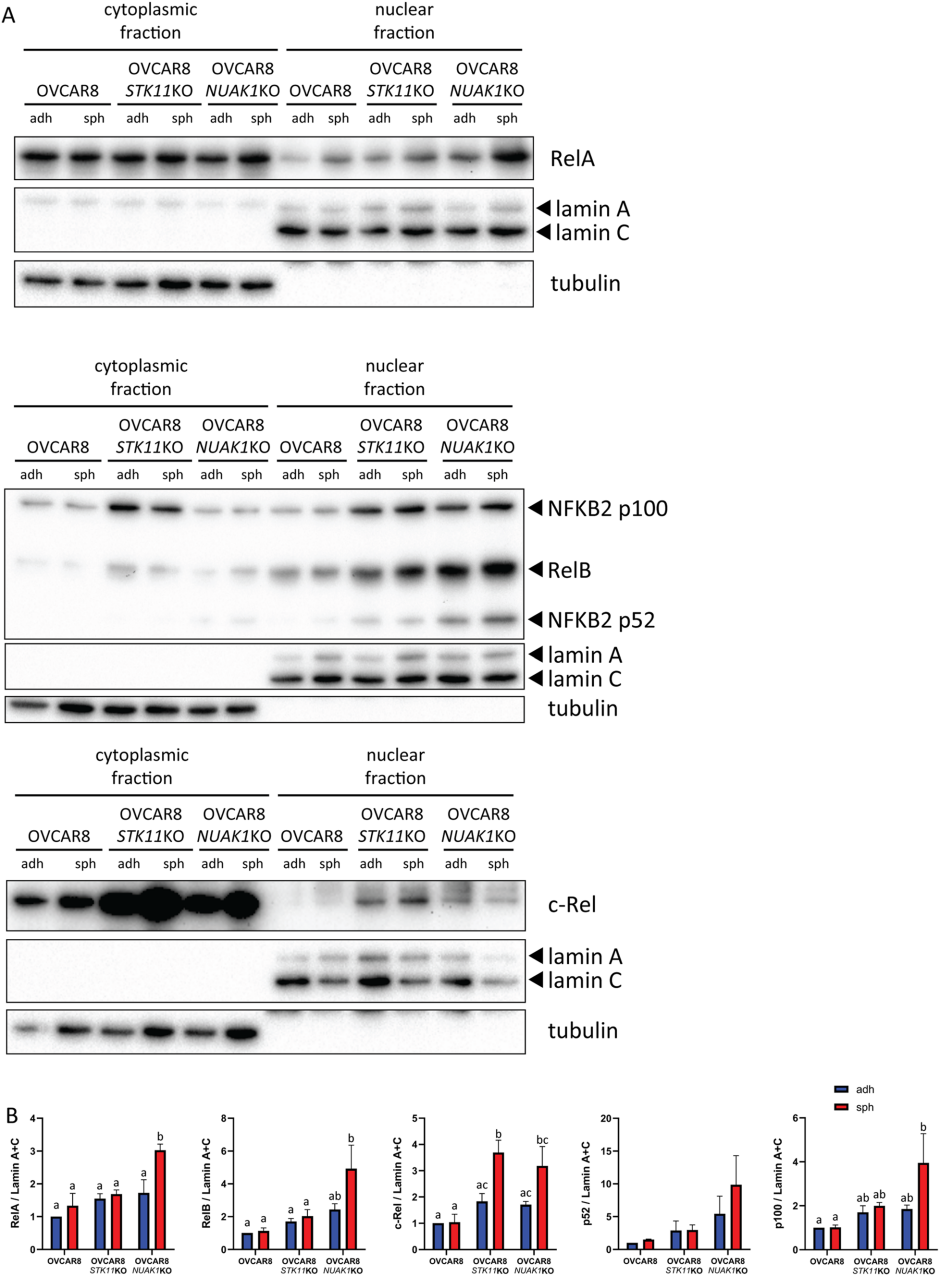
Increased nuclear abundance of NF-κB family transcription factors in OVCAR8-*STK11*KO and OVCAR8-*NUAK1*KO spheroid cells. (**A**) Representative immunoblots of cytoplasmic and nuclear abundance of NF-κB transcription factors in OVCAR8, OVCAR8-*STK11*KO and OVCAR8-*NUAK1*KO cells grown in adherent (adh) and spheroid (sph) culture conditions. Tubulin and lamin A/C were used as controls for cytoplasmic and nuclear fractions, respectively. (**B**) Densitometric analysis of NF-κB transcription factor protein expression. Data are shown as fold-change relative to parental adherent controls shown in blue (adherent culture) or red (spheroid culture). Statistical analysis was performed by two-way ANOVA and groups were compared by Tukey’s multiple comparisons test with alpha set to 0.05, performing all pairwise comparisons; n ≥ 3. Groups with different letters represent statistically significant differences. Error bars indicate standard error of the mean (S.E.M.).

Transcription factors of the NF-κB family reside predominantly in the cytoplasm due to interactions with inhibitory subunits to keep them in an inactive state^49^. Upon pathway activation, NF-κB transcription factors are released from inhibition and translocate to the nucleus to activate specific target gene expression. Therefore, to further assess NF-κB pathway activation, we evaluated transcription factor abundance in cytoplasmic and nuclear fractions. Nuclear abundance of RelA, RelB, and c-Rel was significantly elevated in OVCAR8 spheroids lacking NUAK1 as compared with parental cell spheroids, whereas c-Rel alone was increased in OVCAR8-*STK11*KO spheroids (Fig. 3A, B). Proteolytic processing of p100 to p52 is required for non-canonical NF-κB signalling activation^50^. We observed a significant increase in p52 whole-cell abundance in both OVCAR8-*STK11*KO and OVCAR8-*NUAK1*KO spheroids as compared with controls (Supplementary Fig. S3A,B). These results indicate that canonical and non-canonical NF-κB signalling are elevated due to LKB1 or NUAK1 loss in EOC spheroids, but levels of each transcription factor may be different in each context. No significant differences were seen for RelA, RelB, or c-Rel nuclear localization in native OVCAR8 spheroids as compared with adherent cells (Fig. 3A, B), suggesting that enhanced NF-κB target gene expression in OVCAR8 spheroids may be the result of RelA (S536) phosphorylation.

### Activated NF-κB signalling promotes spheroid cell viability in the context of NUAK1 loss

**Figure 4:**
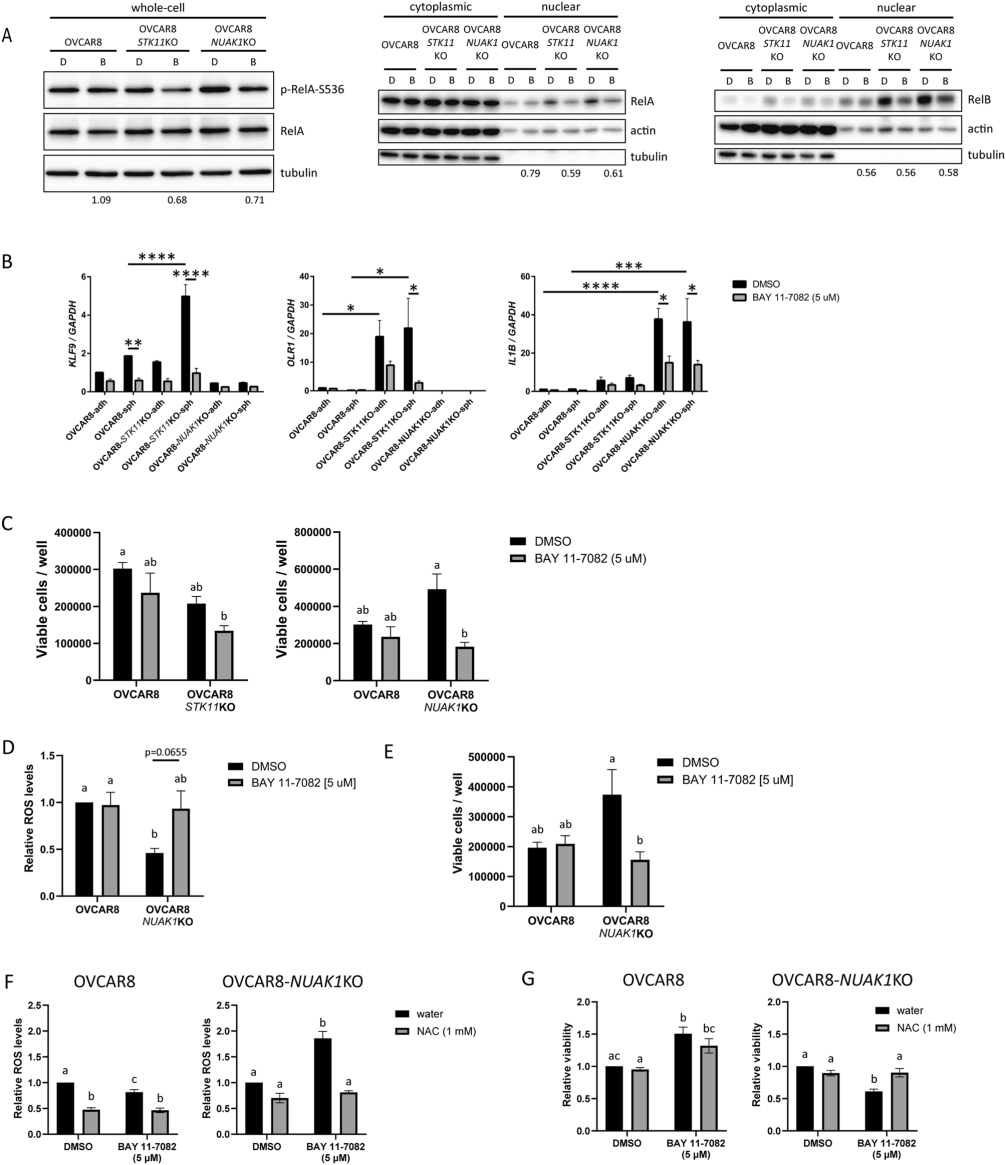
BAY 11-7082 attenuates NF-κB signalling and induces a ROS-dependent decrease in OVCAR8-*NUAK1*KO EOC spheroid cell viability. (**A**) Representative immunoblots of spheroid cell lysates showing total abundance of p-RelA (S536), and cytoplasmic and nuclear abundance of RelA and RelB in OVCAR8, OVCAR8-*STK11*KO and OVCAR8-*NUAK1*KO spheroid cells treated with DMSO (**D**) or 5 μM BAY 11-7082 (**B**). Values under blot images indicate fold-change relative to DMSO controls (p-RelA (S536) normalized to total RelA; RelA and RelB normalized to actin). (**B**) Expression of selected NF-κB target genes in OVCAR8, OVCAR8-*STK11*KO and OVCAR8-*NUAK1*KO cells treated with DMSO or 5 μM BAY 11-7082, as measured by RT-qPCR. (**C**) Viability of OVCAR8, OVCAR8-*STK11*KO and OVCAR8-*NUAK1*KO cells cultured as spheroids for five days with DMSO or 5 μM BAY 11-7082. Cell viability was measured using Trypan blue exclusion of dissociated spheroid cells. (**D**) ROS levels in OVCAR8 and OVCAR8-*NUAK1*KO cells cultured as spheroids for seven days with DMSO or 5 μM BAY 11-7082. Data are shown as ROS-Glo luminescence values normalized to CyQuant fluorescence values, and fold-change relative to OVCAR8 DMSO-treated control. (**E**) Viability of OVCAR8 and OVCAR8-*NUAK1*KO cells cultured as spheroids for seven days with DMSO or 5 μM BAY 11-7082. Cell viability was measured using Trypan blue exclusion of dissociated spheroid cells. (**F**) ROS levels in OVCAR8 and OVCAR8-*NUAK1*KO cells cultured as spheroids for seven days with DMSO or 5 μM BAY 11-7082 with or without 1 mM NAC. Data are shown as ROS-Glo luminescence values normalized to CyQuant fluorescence values, and fold-change relative to vehicle-treated controls. (**G**) Cell viability for OVCAR8 and OVCAR8-*NUAK1*KO cells cultured as spheroids for seven days with DMSO or 5 μM BAY 11-7082, with or without 1 mM NAC. Data are shown as CyQuant fluorescence values normalized to DMSO-water-treated controls. For (**B**–**G**), statistical analysis was performed using two-way ANOVA and groups were compared by Tukey’s multiple comparisons test with alpha set to 0.05, performing all pairwise comparisons. For (**B**), *p ≤ 0.05, **p ≤ 0.01, ***p ≤ 0.001, ****p ≤ 0.0001; n ≥ 3. For (**C**–**G**), groups with different letters represent statistically significant differences; n ≥ 3. For all bar graphs, error bars indicate standard error of the mean (S.E.M.).

LKB1 and its direct substrate NUAK1 have been implicated in mediating cellular adaptation to stress^4,23,51^. Therefore, ablation of either of these kinases in EOC cells may necessitate additional stress responses to maintain cell viability. Given our results, as well as evidence provided by others that NF-κB signalling is activated as an adaptive response due to LKB1 loss^27,28,41^, we sought to ablate NF-κB inflammatory signalling in our system. BAY 11-7082 is a small-molecule inhibitor against IKK1 and IKK2^52,53^, which are required for non-canonical and canonical NF-κB pathways, respectively^54^. We assessed nuclear abundance of RelA and RelB, as they represent primary transcription factors for canonical and non-canonical NF-κB signalling, respectively. Indeed, BAY 11-7082 treatment (Supplementary Fig. S4) decreased RelA and RelB nuclear abundance in OVCAR8, OVCAR8-*STK11*KO and OVCAR8-*NUAK1*KO spheroids (Fig. 4A). BAY 11-7082 also substantially decreased p-RelA (S536) in OVCAR8-*STK11*KO and OVCAR8-*NUAK1*KO spheroids (Fig. 4A). In addition, we confirmed that BAY 11-7082 treatment blocked the upregulation of several NF-κB target genes in our experimental model: i.e., *KLF9* in OVCAR8 and OVCAR8-*STK11*KO spheroids, *OLR1* in OVCAR8-*STK11*KO spheroids, and *IL1B* in both adherent and spheroid *NUAK1*KO cells (Fig. 4B).

To address our idea that increased NF-κB signalling promotes stress-induced cell survival in EOC spheroids, we assessed the effect of NF-κB blockade on EOC spheroid viability. Intriguingly, BAY 11-7082 significantly decreased viable cell number in OVCAR8-*NUAK1*KO spheroids compared to DMSO-treated controls, but this effect was not observed in either OVCAR8 or OVCAR8-*STK11*KO spheroids (Fig. 4C). NUAK1 may be required for protection from oxidative stress as observed in colorectal cancer cells^23^, and NF-κB-mediated antioxidant gene expression occurs in EOC cells^55^. Thus, increased NF-κB signalling in OVCAR8-*NUAK1*KO cells may be required to abrogate elevated oxidative stress due to ROS. Indeed, OVCAR8-*NUAK1*KO spheroids had significantly reduced ROS levels as compared with OVCAR8 spheroids, but this was reversed when treated with BAY 11-7082, which also decreased cell viability (Fig. 4D, E). In order to determine whether ROS contribute to the decreased viability observed in BAY 11-7082-treated OVCAR8-*NUAK1*KO spheroids, we co-treated with the antioxidant N-acetylcysteine (NAC). Treatment with BAY 11-7082 significantly increased ROS levels and decreased viability in OVCAR8-*NUAK1*KO spheroid cells, but these effects were reversed by co-treatment with NAC (Fig. 4F, G). These results indicate that in OVCAR8-*NUAK1*KO spheroid cells, BAY 11-7082 decreases viability in a ROS-dependent manner. This is consistent with the idea that in EOC spheroid cells lacking NUAK1, elevated NF-κB signaling is protective against oxidative stress. Since the importance of NF-κB signalling activity appeared to be crucial for EOC cell viability in the context of NUAK1 loss, we focused our subsequent analysis using *NUAK1*KO cells.

Given that nuclear abundance for both RelA and RelB is significantly elevated in OVCAR8-*NUAK1*KO spheroids cells (Fig. 3A, B), we sought to determine whether knockout of NUAK1 in EOC cells specifically necessitates canonical or non-canonical NF-κB signalling to promote spheroid cell viability. We performed transient siRNA-mediated knockdown of *RELA* and *RELB* (Supplementary Fig. S5), which are the primary mediators of transcription in the canonical- and non-canonical NF-κB pathways, respectively. Knockdown was confirmed by immunoblot analysis in lysates prior to seeding cells for spheroids, and spheroid cell viability was assessed after seven days (Supplementary Fig. S5A). Decreased NF-κB activity was confirmed by assessing *IL1B* and *IL1A* transcription, two NF-κB target genes with observed upregulation in OVCAR8-*NUAK1*KO cells (Supplementary Fig. S5B).

Knockdown of RELA and RELB, alone or in combination, did not decrease OVCAR8-*NUAK1*KO spheroid cell viability (Supplementary Fig. S5C), indicating that additional IKK- and/or NF-κB-associated signalling mechanisms may be involved in promoting OVCAR8-*NUAK1*KO spheroid cell viability.

### NUAK1 loss increases NF-κB signalling in fallopian tube epithelial cells

**Figure 5:**
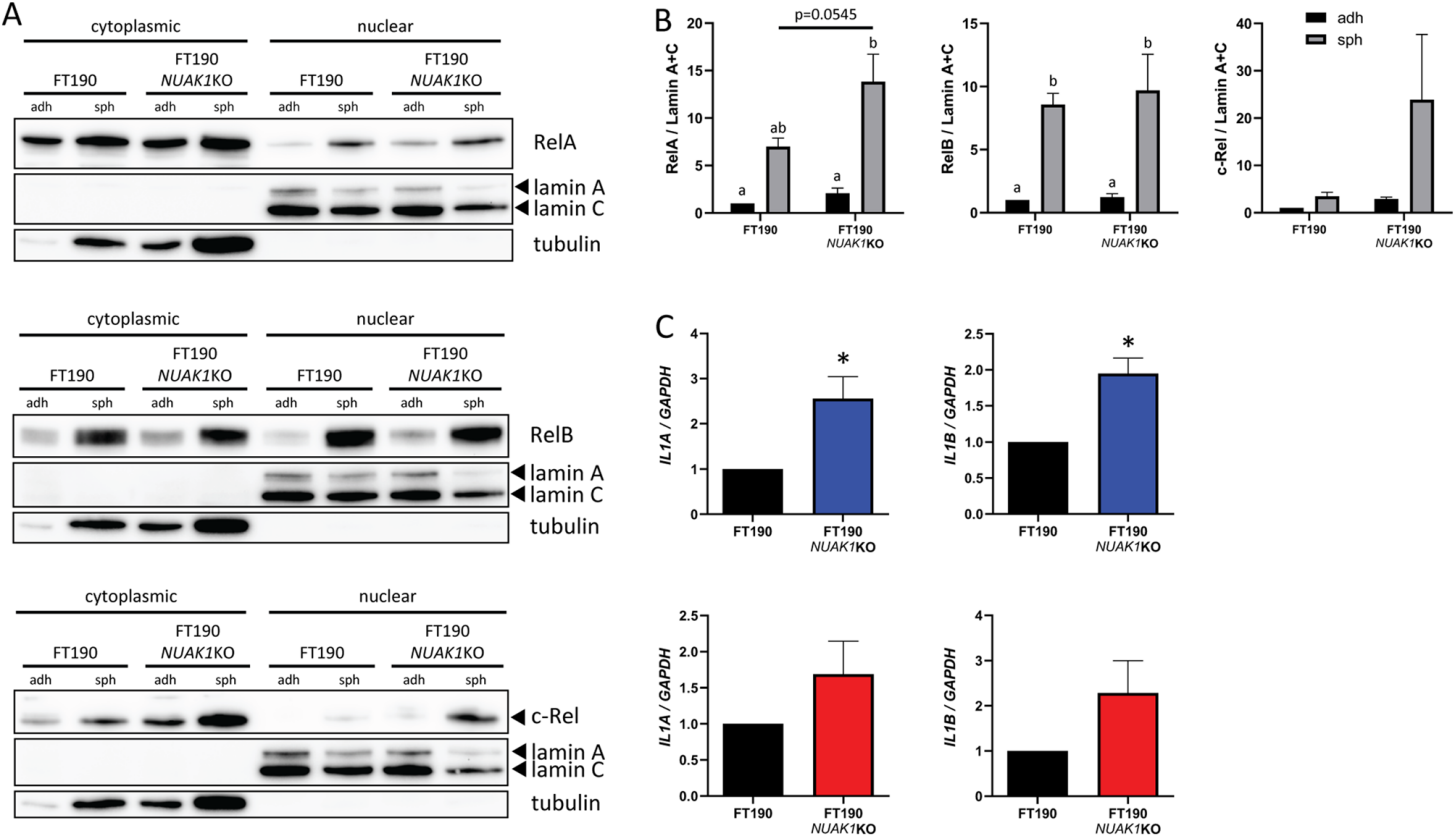
Nuclear abundance of NF-κB family transcription factors is increased in FT190 spheroid cells. (**A**) Representative immunoblots of cytoplasmic and nuclear abundance of NF-κB transcription factors in FT190 and FT190-*NUAK1*KO cells grown in adherent (adh) and spheroid (sph) culture conditions. Tubulin and lamin A/C were used as controls for cytoplasmic and nuclear fractions, respectively. (**B**) Densitometric analysis of NF-κB transcription factor protein expression shown as fold-change relative to parental cells in adherent culture. Statistical analysis was performed using two-way ANOVA followed by Tukey’s multiple comparisons test with alpha set to 0.05, performing all pairwise comparisons; n = 3. Letter labels indicate statistically significant differences between groups. (**C**) Fold change in expression of selected NF-κB target genes in FT190-*NUAK1*KO cells relative to FT190 control cells as determined by RT-qPCR (adherent FT190-*NUAK1*KO cells shown in blue, and FT190-*NUAK1*KO spheroid shown in red). Statistical analysis was performed by unpaired, two-tailed Student’s *t*-test (*p ≤ 0.05; n = 3). For all bar graphs, error bars indicate standard error of the mean (S.E.M.).

Fallopian tube secretory epithelial cells (FTSEC) are considered the major cell-of-origin for HGSOC^56–58^. To determine whether increased NF-κB signalling due to NUAK1 ablation is specific to HGSOC cells or may also be a property of pre-neoplastic FTSEC cells, we performed CRISPR/Cas9-mediated knockout of *NUAK1* in FT190 cells, an immortalized human fallopian tube secretory epithelial cell line^59^. We observed a significant increase in RelA nuclear abundance in FT190-*NUAK1*KO, but not parental FT190, spheroids as compared with adherent cells (Fig. 5A, B). RelB nuclear abundance was elevated in spheroids as compared with adherent cells for both FT190-*NUAK1*KO cells and FT190 controls (Fig. 5A, B). Moreover, a trend towards increased nuclear abundance of RelA in FT190-*NUAK1*KO spheroids as compared with FT190 control spheroids was observed (p = 0.0545; Fig. 5B). Further to this, there was a significant increase in expression of two representative NF-κB target genes, *IL1A* and *IL1B*, in FT190-*NUAK1*KO adherent cells as compared with FT190 controls (Fig. 5C). These differences were not statistically significant in spheroids, however, perhaps due to the already elevated nuclear abundance of NF-κB transcription factors in both FT190 and FT190-*NUAK1*KO spheroid cells (Fig. 5A, B). These results suggest that NUAK1 loss has the capacity to enhance NF-κB signalling even in precursor cells of HGSOC.

### Elevated nuclear RelB expression in OVCAR8-*NUAK1*KO xenograft tumours

**Figure 6:**
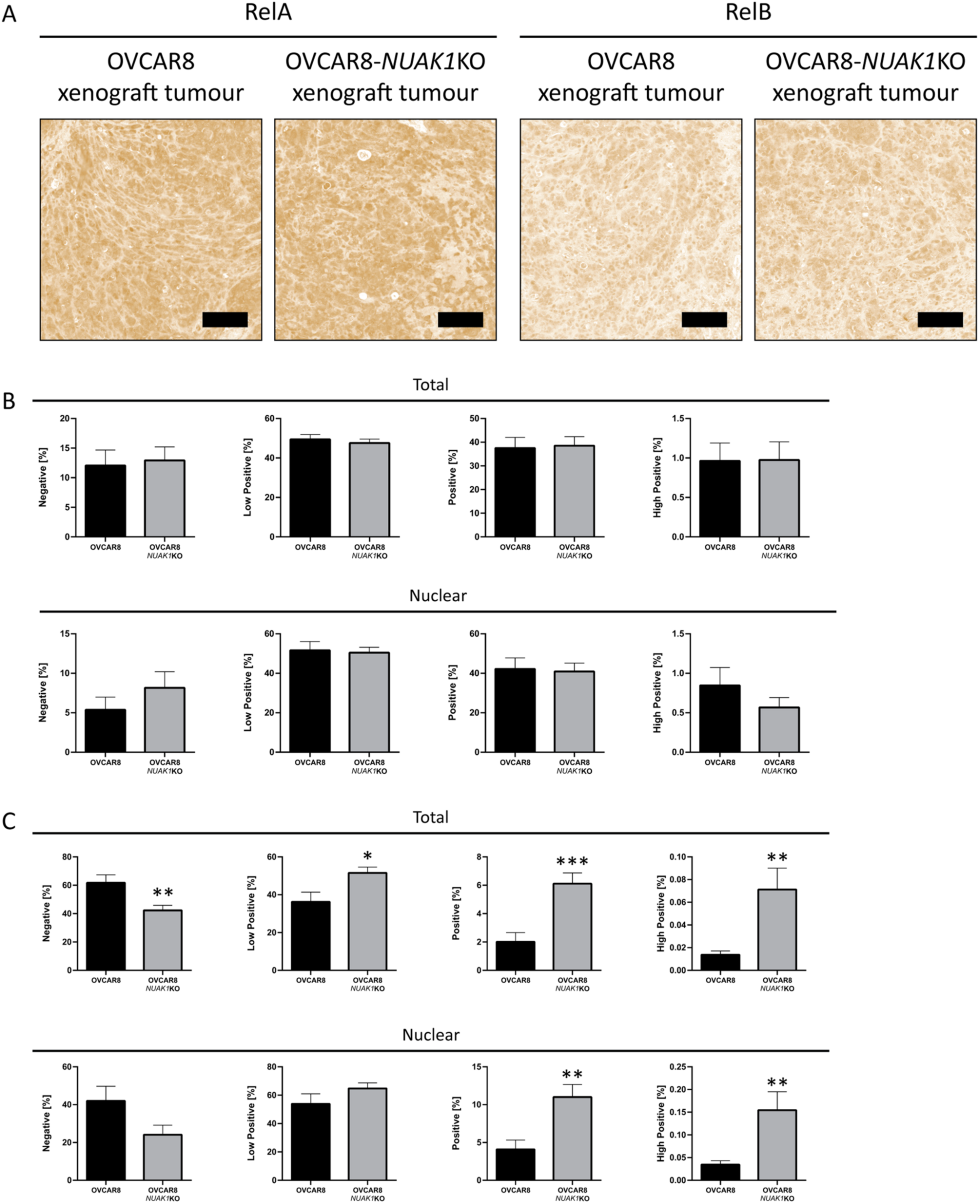
Enhanced nuclear staining of RelB in OVCAR8-*NUAK1*KO xenograft tumours. (**A**) Representative immunohistochemistry DAB channel images for RelA and RelB in xenograft tumours using OVCAR8 and OVCAR8-*NUAK1*KO cells. Scale bars represent 100 μm. (**B**, **C**) Quantification of DAB staining intensity for RelA (**B**) and RelB (**C**) as determined by the *IHC Profiler* ImageJ plugin. Data are shown as the percentage of pixels classified as “Negative”, “Low Positive”, “Positive”, and “High Positive” for the full image (Total) or nuclear regions as defined by hematoxylin staining (Nuclear). OVCAR8-*NUAK1*KO were compared with OVCAR8 by unpaired, two-tailed Student’s *t*-test (*p ≤ 0.05, **p ≤ 0.01, ***p ≤ 0.001; n ≥ 10 tumour nodules per group). Error bars indicate standard error of the mean (S.E.M.).

Spheroids are key contributors to EOC metastasis via characteristics that favour dissemination, such as evasion of anoikis^60^ and resistance to chemotherapy, and they have transcriptional profiles that differ from primary and secondary adherent tumours^61^. To determine whether enhanced NF-κB signalling due to NUAK1 loss is a spheroid-specific property or is present in solid tumours as well, we assessed nuclear localization of RelA and RelB by immunohistochemical staining in mouse intraperitoneal tumour xenografts generated as part of a previous study^20^. We observed significantly increased positive nuclear staining for RelB in OVCAR8-*NUAK1*KO xenograft tumours as compared with OVCAR8 tumours, however no significant differences in RelA staining were observed (Fig. 6A–C). These results indicate that activated NF-κB signalling due to NUAK1 loss in spheroids is sustained in metastatic tumour deposits, but the relative contribution of canonical and non-canonical NF-κB signalling may differ in each context during spread of disease. This is consistent with the dissimilar kinetics that have been reported for the canonical and non-canonical NF-κB signalling pathways, which have been described as transient and persistent, respectively^50,62–64^. As such, while both canonical and non-canonical NF-κB signalling are enhanced in OVCAR8-*NUAK1*KO spheroids, non-canonical signaling may represent a lasting response in tumours that persists beyond induction and attenuation of canonical signalling in spheroids.

Next, we sought to determine whether decreased LKB1-NUAK1 activity is associated with altered NF-κB signalling in human ovarian tumours. We ranked ovarian tumour samples published in The Cancer Genome Atlas using a 16-gene signature associated with LKB1 loss^65^ and performed Gene Set Variation Analysis^66^ (Supplementary Fig. S6A). The top 33% of samples (analogous to low LKB1 activity) had significantly increased HALLMARK_TNFA_SIGNALING_VIA_NFKB GSVA scores compared to the bottom 33% (Supplementary Fig. S6B), consistent with the idea that loss of LKB1 in EOC cells enhances NF-κB signalling. In contrast, the bottom 33% of samples based on *NUAK1* mRNA (analogous to low NUAK1 expression) had significantly lower HALLMARK_TNFA_SIGNALING_VIA_NFKB GSVA scores compared to the top 33% (Supplementary Fig. S6C,S6D). This may indicate that NUAK1-NF-κB crosstalk differs between tumours and spheroid or xenograft models of EOC, but may also reflect the possibility that *NUAK1* mRNA expression is not a reliable surrogate for NUAK1 activity.

## Discussion

We previously reported that depletion of LKB1 in EOC cells decreases metastatic potential in suspension culture and orthotopic mouse xenograft models of metastasis^16,43^. Furthermore, our recent work on the direct LKB1 substrate NUAK1 revealed key roles in regulating adhesion molecules and promoting metastatic potential in EOC^20^. In the current study, we present intriguing new evidence that NF-κB signalling is activated in EOC spheroid cells which is further enhanced by ablation of LKB1-NUAK1 function. Interestingly, there may be differences between LKB1 and NUAK1 requirements for NF-κB signalling regulation of either canonical or non-canonical pathways. Lastly, specific loss of NUAK1 increases ROS levels and necessitates enhanced NF-κB signalling for the maintenance of EOC spheroid cell viability.

EOC spheroids have been widely implicated in promoting spread of disease and supporting the emergence of chemoresistance^67^. NF-κB signalling promotes multicellular tumour spheroid formation and growth in multiple cancer cell line models^68^. For example, RelA promotes cell proliferation while RelB supports viability of tumour-initiating cells, and both act to promote spheroid formation^69^. Herein, we also show enhancement of an NF-κB transcriptional signature in EOC spheroids with increased p-RelA (S536) in these structures. Proinflammatory NF-κB signalling in EOC is typically associated with poor patient outcomes, owing to the protection it confers from chemotherapy and immune surveillance. Furthermore, NF-κB signalling is recognized as an important stress response pathway in EOC, protecting against oxidative stress, anoikis, and other stressors^55,70,71^. As such, enhanced NF-κB signalling in spheroids may be an adaptation that promotes metastasis in EOC. Spheroids, considered a primary vehicle for EOC cell transit during metastasis, may contribute to elevated proinflammatory cytokines found in ascites associated with immunosuppression.

Our previous studies have implicated a crucial role for intact LKB1-NUAK1 signalling in promoting spheroid formation, cell viability, and metastatic potential^16,20,43^. Further investigation of the altered transcriptome in EOC spheroids lacking either LKB1 or NUAK1 led to our discovery of enhanced NF-κB signalling pathway activation. Several other reports support the concept that LKB1 can repress NF-κB signalling. Depletion of LKB1 is associated with enhanced NF-κB signalling in B cells^41^ and skeletal muscle^28^. LKB1 can interact with IKKβ leading to repression of NF-κB signalling in macrophages^27^. There are fewer reports demonstrating cross-talk regulation between NUAK1 and NF-κB signalling. NUAK1 may activate the canonical NF-κB pathway via phospho-RelA in the lung cancer cell line A549^72^. However, our results demonstrate NUAK1 loss enhances NF-κB signalling in the high-grade serous cell line OVCAR8, as well as in the precursor cell model FT190. It is well-established that NF-κB signalling is activated by numerous cellular stressors^46^ and, amongst its many functions, can promote cell survival^73^. Given that NUAK1 may serve an important role in the response to metabolic^74^ and oxidative stress^23^, NUAK1 ablation may subject spheroid cells to increased levels of these particular stresses. Indeed, we observed that OVCAR8-*NUAK1*KO spheroid cells require enhanced NF-κB signalling to deal with ROS and maintain spheroid cell viability. Further to these findings, we observed that immunohistochemical staining for nuclear RelB, but not RelA, was elevated in xenografts of OVCAR8-*NUAK1*KO cells, indicating sustained activation of non-canonical NF-κB signalling in metastatic implants. Taken together, our findings suggest that loss of NUAK1 necessitates inflammatory stress response signalling in early precursor lesions through to late-stage metastasis during a broad window of disease progression.

In many cell types, the oxidative stress response is primarily mediated by the NRF2 pathway, which drives expression of antioxidant genes^75^. In colorectal cancer cells, NUAK1 is activated by oxidative stress and is required for nuclear import of NRF2 and subsequent induction of antioxidant response genes^23^. Perhaps, in our system, loss of NUAK1 forces EOC spheroid cells to adopt NF-κB mediated mechanisms to respond to elevated oxidative stress as an alternative to NRF2. We observed that *KLF9* expression is significantly decreased while expression of *IL1B* and several other pro-inflammatory cytokines are significantly increased due to NUAK1 loss. These results would suggest impaired NRF2 signalling since ROS-induced expression of *KLF9* is known to occur via the NRF2 pathway^76^, while expression of *IL1B* and other pro-inflammatory cytokines is repressed by NRF2^42^. Indeed, treatment of OVCAR8-*NUAK1*KO spheroids with the IKK inhibitor BAY 11-7082 increased ROS levels, which is consistent with a recent report that NF-κB-associated expression of antioxidant genes protects ovarian cancer cells from oxidative stress^55^. Thus, enhancement of NF-κB signalling may be an adaptive response to impaired ROS clearance in NUAK1-deleted spheroid cells. This idea is further supported by our finding that co-treatment with the antioxidant NAC decreased ROS and blocked the ability of BAY 11-7082 to decrease OVCAR8-*NUAK1*KO spheroid viability, suggesting that activation of NF-κB signalling is protective against oxidative stress in a NUAK1-deleted model of EOC metastasis.

The results in this present study were largely driven by the use of cell lines in which the *STK11* and *NUAK1* genes were inactivated using CRISPR genome editing technology. However, our studies have a potential limitation since we compare *STK11*- and *NUAK1*-knockout cells to their respective parental cell lines, but these control cells were not taken thorough the same CRISPR processing in parallel. Since off-target Cas9 nuclease activity has been described^77^, appropriate controls are warranted to minimize the phenotypic consequences of this potential limitation. The knockout lines we used in this present study had been generated and characterized previously^16,20^, and at that time we used pooled populations of multiple knockout clones to minimize individual artifacts that may have arisen due to random Cas9 activity. While our chosen approach does not preclude clone-specific, off-target effects, we expect their net impact to be reduced significantly as a result.

While ROS play important roles in cell signalling, excessive levels damage macromolecules critical to cell survival and induce multiple types of cell death. As such, ROS induction has been considered as a therapeutic strategy for some cancers^78^. If therapeutic strategies were to be employed targeting NUAK1 in advanced EOC, particularly within the harsh environment of malignant ascites^79–81^, combination with antioxidant inhibitors would be required for maximum efficacy. Our findings warrant further investigation to determine whether targeting NUAK1 can enhance the effectiveness of therapeutic strategies that benefit from ROS induction in cancer cells.

## Materials and methods

### Antibodies and reagents

Antibodies against p-RelA-S536 (#3033; 1:1000), RelA (#8242; 1:1000), RelB (#4922, 1:2000; IHC: #10544), c-Rel (#4727; 1:1000), NF-κB2 (#3017; 1:1000), LKB1 (#3050; 1:1000), and NUAK1 (#4458; 1:1000) were purchased from Cell Signalling Technology (Danvers, MA). Antibodies against lamin A/C (MAB3211; 1:500) and actin (A2066; 1:20000) was purchased from Millipore (Temecula, CA, USA). Antibody against tubulin (T5168; 1:20000) was purchased from Sigma. HRP-conjugated antibodies against mouse IgG (NA931; 1:10000) and rabbit IgG (NA934; 1:10000) were purchased from Cytiva. All antibodies were diluted in tris-buffered saline-Tween 20 containing 5% bovine serum albumin, with the exception of NUAK1 (tris-buffered saline-Tween 20 containing 5% nonfat milk). BAY 11-7082 was purchased from Cayman Chemical (cat. 10010266; Ann Arbor, MI, USA). ROS-Glo H_2_O_2_ Assay was purchased from Promega (cat. G8820).

### Generation of *STK11*KO and *NUAK1*KO cell lines

Generation of OVCAR8-*STK11*KO^16^ and OVCAR8-*NUAK1*KO^20^ cell lines has been previously described. For FT190-*NUAK1*KO cells, two independent 20-nucleotide guide sequences targeting the *NUAK1* gene 5′-GTGGC GGGGG ACCGC CCCGA-3′ (site 1) and 5′-GGGTC TCCTG CAGCT CGTAG CGG-3′ (site 2) were selected using the CRISPR Design Tool (http://tools.genome-engineering.org). Complementary oligonucleotides 5′-CACCG TCGGG GCGGT CCCCC GCCAC-3′ and 5′-CACCG GGGTC TCCTG CAGCT CGTAG-3′ for site 1 and 5′-CACCG GGGTC TCCTG CAGCT CGTAG-3′ and 5′-AAACC TACGA GCTGC AGGAG ACCCC-3′ for site 2 (Sigma-Genosys) were annealed and ligated into the BbsI-digested restriction endonuclease site of pSpCas9(BB)-2A-Puro plasmid^82^ (gift from Dr. F. Dick, Western University) to generate the pSpCas9-sgNUAK1-1 and -2 plasmids. Cells were seeded at 200,000 cells/well into 6-well plates and transfected with 0.5 μg each of pSpCas9-sgNUAK1 plasmids using LipofectAMINE2000 (Invitrogen) according to the manufacturer’s instructions. Media containing 1 μg/mL puromycin was replaced the following day, and cells were treated for one day. After growth recovery, the cells were trypsinized, counted, and seeded into 96-well plates to perform limiting dilution subcloning of *NUAK1*-knockout cells. Single colonies were expanded for protein isolation and the confirmation of NUAK1 loss by immunoblotting. Four clones lacking NUAK1 protein expression were identified by immunoblotting and pooled to generate a mixed population.

For experiments in Figs. 1, 2, 3, 4A–C and 6 and Supplementary Figs. S1A, S3, S4A–B, OVCAR8-*NUAK1*KO clone 62 was used. For experiments in Fig. 4D–G and Supplementary Figs. S1C, S4C–D, and S5, a mixed population containing equal proportions of 3 OVCAR8-*NUAK1*KO clones (50, 62, 63) were used to confirm that results were not clone-specific.

### Cultured cell lines

OVCAR8, OVCAR8-*STK11*KO, OVCAR8-*NUAK1*KO, HeyA8, and HeyA8-*STK11*KO cell lines were cultured in RPMI-1640 (Wisent). iOvCa147, iOvCa147-*STK1*1KO, FT190, and FT190-*NUAK1*KO cell lines were cultured in DMEM/F12 (Life Technologies). For all cell lines, growth medium was supplemented with 10% fetal bovine serum. OVCAR8 and HeyA8 cells were obtained from the American Type Culture Collection; iOvCa147 cells were generated by our research group as previously described^83^. Adherent cells were maintained on tissue culture-treated polystyrene (Sarstedt, Newton, NC, USA). Spheroids were maintained in Ultra-Low Attachment (ULA) cluster plates (Corning, NY, USA). The immortalized human fallopian tube secretory epithelial cell line FT190^59^ was provided by R. Drapkin (University of Pennsylvania, Philadelphia, PA). All cell lines were authenticated by short tandem repeat analysis performed by The Centre for Applied Genomics (The Hospital for Sick Children, Toronto, ON, Canada) and routinely tested for mycoplasma using the Universal Mycoplasma Detection Kit (30-1012K; ATCC).

### Microarray analysis

Cells were seeded into 10 cm tissue culture-treated dishes (1 × 10^6^ cells in 12 mL; 1 dish per sample) or 6-well ULA plates (5 × 10^5^ cells in 5 mL; 3 wells per sample). Spheroids were harvested for RNA purification 24 h post-seeding; adherent cells were harvested 48 h post-seeding. RNA was collected as described in *RNA collection and purification*, and two 100 ng/μL dilutions in nuclease-free water were prepared for each sample: 3 μL for Bioanalyzer analysis to confirm acceptable RNA prep quality, and 10 μL for Clariom S microarray analysis. Dilutions were frozen on dry ice and shipped to The Centre for Applied Genomics (The Hospital for Sick Children, Toronto, ON, Canada) for further processing. Data analysis and export was performed using the *Transcriptome Analysis Console 4.0.1* software package (Thermo Fisher).

### RNA collection and purification

RNA was extracted using the RNEasy Spin Column kit (cat. 74104; Qiagen) according to the manufacturer’s protocol, with the optional DNaseI (cat. 79254; Qiagen) treatment. Adherent cells were collected by aspirating medium and scraping cells into 600 μL Buffer RLT and stored at − 80 °C until processing. Spheroids were pelleted at 800 g, 4 °C, medium was aspirated, and cells were lysed in 350 μL Buffer RLT and stored at − 80 °C until processing. RNA concentration, A_260/280_, and A_280/230_ were determined using a NanoDrop One Microvolume UV–Vis Spectrophotometer (Thermo Scientific).

### Gene set enrichment analysis

Sample signals were exported using the *Transcriptome Analysis Console 4.0.1* software package and formatted according to GSEA guidelines (https://software.broadinstitute.org/cancer/software/gsea/wiki/index.php/Data_formats) for *Text file format for expression dataset*. Phenotype labels were generated according to the *Categorical class file format*. The following GSEA (ver. 3.0) parameters were used: gene sets database, “h.all.v6.2.symbols.gmt”; permutation type, “gene_set”; number of permutations, 1000; collapse/remap to gene symbols, collapse; chip platform, “Clariom_S_Human.r1.chip”, enrichment statistic, weighted; metric for ranking genes, Signal2Noise; gene list sorting mode, real; gene list ordering mode, descending; maximum gene set size, 500; minimum gene set size, 15; collapsing mode for probe sets, max_probe; normalization mode, meandiv; randomization mode, no_balance; omit symbols with no match, true; median for class metrics, false; seed for permutation, timestamp. For identifying significantly enriched gene sets, p-value and false discovery rate cutoffs of 0.05 and 0.25, respectively, were used. Venn diagrams in Fig. 1A, B and Supplementary Fig. S2A were generated using the matplotlib-venn package (https://github.com/konstantint/matplotlib-venn).

### Quantitative reverse transcription PCR

cDNA synthesis was performed using the High Capacity cDNA Reverse Transcription Kit (Thermo Fisher) according to the manufacturer’s protocol using 2000 ng RNA per reaction, yielding a final volume of 20 uL per reaction. Reactions were incubated in a MyCycler thermocycler (BioRad) using the following settings: 25 °C for 10 min, 37 °C for 120 min, 85 °C for 5 min, hold at 4 °C until sample retrieval. Each reaction was then diluted with an equal volume of nuclease-free water. qPCR was performed using the Brilliant II SYBR Green QPCR Master Mix (Agilent Technologies) according to the manufacturer’s protocol scaled to a 10 μL reaction volume. Reactions were assembled in a 96-well plate, sealed with adhesive plastic film, and then centrifuged for 20 s to remove bubbles. Cycling was performed in a QuantStudio 3 RT-PCR System (Thermo Fisher) using built-in settings for SYBR Green Chemistry, Fast mode. Data analysis was performed using *QuantStudio Design and Analysis Software 1.4.3*. Fold-change values relative to controls were calculated using using the 2^−ΔΔC^_T_ method^84^. Primer sequences (Supplementary Table S2) were obtained from https://www.origene.com and purchased from Invitrogen.

### Preparation of whole-cell, cytoplasmic and nuclear lysates

For assessment of total p-RelA (S536), day 3 whole-cell lysates were generated from adherent cells cultured at a density of 0.5–3 × 10^6^ cells in 13 mL medium (10 cm dish), or spheroid cells cultured at a density of 1–3 × 10^6^ cells in 6 mL medium (35 mm ULA well). For assessment of NF-κB transcription factor nuclear abundance, day 1 whole, cytoplasmic and nuclear lysates were generated from adherent cells cultured at a density of 3.5–4.5 × 10^6^ cells in 13 mL medium (10 cm dish), or spheroid cells cultured at a density of 1.25–1.5 × 10^6^ cells in 5 mL medium (35 mm ULA well). Seeding numbers were chosen to obtain acceptable protein yields for each cell line.

#### Whole-cell lysates

Adherent cells grown in tissue culture-treated plates or dishes were collected by aspirating medium, washing 2 × with cold PBS, and scraping into modified RIPA buffer. Spheroids (at least 1.5 × 10^6^ cells per sample) were collected by transferring the cell suspension into a conical tube on ice, pelleting by centrifugation in a swinging bucket rotor (800×*g* at 4 °C for 4 min), aspirating medium, resuspending in at least 10 mL cold PBS, pelleting, resuspending again in cold PBS, pelleting, and aspiration of PBS. Cell pellets were then lysed in modified RIPA buffer, vortexed, subjected to one freeze–thaw cycle, and clarified by centrifugation (max×*g* at 4 °C for 20 min).

#### Cytoplasmic and nuclear lysates

Adherent cells were washed 2× with cold PBS and scraped into cold PBS using a cell lifter, transferred to a 50 mL conical tube and pelleted by centrifugation in a swinging bucket rotor (800×*g* at 4 °C for 4 min). Spheroids (at least 3 × 10^6^ cells per sample) were collected into a conical tube and pelleted by centrifugation in a swinging bucket rotor (800×*g* at 4 °C for 4 min), washed 2× in cold PBS, and pelleted by centrifugation. Cell pellets were resuspended in at least 450 μL cold hypotonic lysis buffer and incubated on ice for 50 min, vortexing (max) for 10 s every 10 min. The suspension was then pelleted by centrifugation (max×*g* at 4 °C for 3 min). The supernatant (cytoplasmic lysate; 90% of hypotonic lysis buffer volume used) was transferred to another microcentrifuge tube and left on ice. The remaining supernatant in the original microcentrifuge tube was aspirated. 1 mL of cold wash buffer was added, vortexed for 20 s, pelleted by centrifugation (max×*g* at 4 °C for 1 min), and the wash buffer was aspirated. This wash step was repeated and the pellet was lysed in 100 μL modified RIPA buffer and vortexed to generate the nuclear lysate. Cytoplasmic and nuclear lysates were then subjected to 1 freeze–thaw cycle and clarified by centrifugation (max×*g* for 20 min at 4 °C). *Hypotonic lysis buffer*: 20 mM HEPES (pH 7.4), 1 mM EGTA, 1 mM EDTA, 1 mM DTT, 0.3% Triton X-100, 1 mM Na_3_VO_4_, 10 mM NaF, 1 mM PMSF, 1 × SIGMA*FAST* protease inhibitor cocktail (S8820; Sigma), 10 mM beta-glycerophosphate. *Fraction Wash buffer*: 10 mM HEPES (pH 7.4), 10 mM KCl, 0.1 mM EGTA, 0.1 mM EDTA, 1 × SIGMA*FAST* protease inhibitor cocktail. *Modified RIPA buffer*: 50 mM HEPES (pH 7.4), 150 mM NaCl, 10% glycerol, 1.5 mM MgCl_2_, 1 mM EGTA, 1% Triton X-100, 0.1% SDS, 1 mM Na_3_VO_4_, 10 mM NaF, 1 mM PMSF, 1 × SIGMA*FAST* protease inhibitor cocktail (cat. S8820; Sigma), 10 mM beta-glycerophosphate. Total protein concentration was determined using the Bio-Rad Protein Assay according to manufacturer’s instructions (cat. 5000006; Bio-Rad).

### Immunoblot analysis

Immunoblotting was performed using the Bio-Rad Mini-PROTEAN II Electrophoresis System (Bio-Rad) according to manufacturer’s instructions using gels cast in-house (30% acrylamide/bis solution 37.5:1, cat. #1610158; Bio-Rad). Densitometry was performed using the Image Lab 6.0.1 software package (Bio-Rad).

### Treatment with BAY 11-7082

Cells were seeded into 10 cm tissue culture-treated dishes at a density of 2.5 × 10^6^ cells in a volume of 12 mL. The following day, cells were pre-treated with drug or vehicle: medium was removed by aspiration and replaced with 10 mL medium containing 5 μM BAY 11-7082 or an equivalent v/v DMSO as a vehicle control. The following day, cells were trypsinized, counted and seeded at the required density in medium containing BAY 11-7082 to a final concentration of 5 μM or an equivalent v/v DMSO. Seeding densities: 24-well ULA spheroids, 0.5–1 × 10^5^ cells in 1 mL; 6-well ULA spheroids, 0.5–1.5 × 10^6^ cells in 5 mL; 96-well ULA spheroids, 1 × 10^4^ cells in 80 μL; 10 cm adherent, 1.5–2.5 × 10^6^ cells in 10–12 mL. Cell numbers were chosen to achieve acceptable yields.

### Trypan blue spheroid viability assay

Cells were seeded into 24-well ULA cluster plates (0.5–1 × 10^5^ cells per well in a volume of 1 mL). Spheroids were collected into microcentrifuge tubes on ice and pelleted by centrifugation at 500×*g* for 3 min. Medium was aspirated and the pellet was washed by resuspension in 500 μL PBS (without drawing cells up into pipette tip), pelleted again as described above, resuspended in 50–250 μL trypsin/EDTA, and incubated at 37 °C with gentle agitation every 10 min until aggregates were no longer visible (10–30 min). Trypsin was then inactivated by adding an equal volume of FBS. Trypan Blue dye was added (volume equal to trypsin/EDTA + FBS) prior to counting the sample and gently mixed by pipetting. Cell counting was performed using a TC20 Automated Cell Counter (Bio-Rad).

### CyQUANT viability assay

Spheroids were collected into microcentrifuge tubes on ice using a beveled P200 tip and pelleted at 500×*g* for 5 min at 4 °C. Medium supernatant was aspirated and the pellet was stored at – 80 °C. Relative viability was assessed by CyQUANT Cell Proliferation Assay (cat. C7026; Thermo Fisher) using 2 × CyQUANT GR dye concentration to extend the assay linear detection range as described in the manufacturer’s instructions, blank-subtracted, and used to normalize ROS-Glo luminescence.

### Measurement of ROS levels in EOC spheroids

Cells were pre-treated with BAY 11-7082 or DMSO as described in *Treatment with BAY 11-7082*. 24 h after treatment, cells were trypsinized, counted and seeded into 96-well round-bottom ULA cluster plates at a density of 10,000 cells/well in 80 μL, maintaining the same concentration of DMSO or BAY 11-7082 (5 μM). At d7, ROS levels were assessed using the ROS-Glo H_2_O_2_ Assay (Promega) according to the manufacturer’s instructions. 20 μL of 125 μM H_2_O_2_ substrate was added to each well and returned to the cell culture incubator for 5 h. 100 μL of complete ROS-Glo detection solution was then added to each well and incubated for 20 min at room temperature on a rocker. 175 μL from each well was then transferred to an opaque white 96-well plate, and luminescence was measured using a Synergy H4 plate reader (Biotek; Winooski, VT, USA). After blank subtraction, ROS-Glo luminescence was normalized to CyQUANT signal measured from identically-seeded and -treated replicate wells. For all experiments involving measurement of ROS levels, all cell culture was performed using DMEM/F12 supplemented with 10% FBS instead of RPMI 1640 in order to minimize ROS-Glo H_2_O_2_ Assay background signal. For experiments involving N-acetylcysteine, “no cell” blank controls contained 1/500 water (to perform blank subtraction for spheroid cells treated with 1/500 water) or 1 mM NAC (to perform blank subtraction for spheroid cells treated with 1 mM NAC) to account for differences in background signal associated with NAC.

### Xenograft tumour histology and immunohistochemistry

Previously archived formalin-fixed, paraffin-embedded tumours^20^ were sectioned at a thickness of 5 μm. Hematoxylin/eosin staining was used to visualize tissue architecture, and adjacent sections were immunohistochemically stained to assess RelA and RelB (1:200 dilution), using hematoxylin as a counterstain. Sectioning and staining were performed by the Molecular Pathology Core Facility at Robarts Research Institute (London, Ontario, Canada). Images of stained tumour sections were captured using an Aperio ScanScope slide scanner (Leica).

### Immunohistochemistry quantification and scoring

IHC analysis was performed using the Fiji distribution of ImageJ^85^. Images of individual stained tumour nodules were exported using the ImageScope software package (Leica). Empty space around nodules was erased using the Adobe Photoshop software package. Necrotic tumour regions were identified using the “Trainable Weka Segmentation” plugin^86^, confirmed by visual inspection, and cleared. RelA and RelB staining was then evaluated using the “IHC Profiler” plugin^87^ for the total image, and for nuclear regions of the image (identified by thresholding of the hematoxylin channel).

### Transient knockdown of *RELA* and *RELB*

Cells were seeded in 6-well plates (1.5 × 10^5^ cells/well) and transfected using DharmaFECT1 (1/500 final dilution) as per manufacturer's protocol (Dharmacon) using a final total siRNA concentration of 10 nM. *RELA* ON-TARGETplus SMARTpool (L-003533-00-0020), *RELB* ON-TARGETplus SMARTpool (L-004767-00), or ON-TARGETplus Non-targeting Pool (D-001810-10) were used. 24 h after transfection, 3 mL of medium were added to each well, for a total volume of 5 mL. 48 h later, cells were trypsinized, counted and seeded for experiments. Immunoblot analysis was performed on d0 adherent and d7 spheroid cell lysates to confirm *RELA* and *RELB* knockdown. RT-qPCR was performed in d1 spheroid cell and d2 adherent cell RNA to confirm decreased NF-κB target gene expression.

### Microscopy

Phase-contrast images were captured using a DMI 4000B inverted microscope (Leica). Brightfield images of spheroids treated with BAY 11-7082 and NAC were captured using an IncuCyte S3 Live-Cell Analysis System (Sartorius). Images of immunohistochemically-stained xenograft tumour sections were captured using an Aperio ScanScope slide scanner (Leica).

### Gene set variation analysis

The dataset “TCGA-OV.htseq_fpkm.tsv” corresponding to the “GDC TCGA Ovarian Cancer (OV)” cohort was retrieved from UCSC Xena Browser (https://xenabrowser.net/datapages/?dataset=TCGA-OV.htseq_fpkm.tsv&host=https%3A%2F%2Fgdc.xenahubs.net&removeHub=https%3A%2F%2Fxena.treehouse.gi.ucsc.edu%3A443) on October 10, 2021. Log2(FPKM + 1) values were converted to Fragments Per Kilobase of transcript per Million mapped reads (FPKM). Ensembl IDs were converted to gene symbols using the AnnotationDbi^88^ package. Expression values for the 16 genes in a transcriptional signature associated with LKB1 loss^65^ were standardized across tumour samples and averaged to calculate a LKB1-loss score for every sample in the dataset. The samples were then ranked by LKB1-loss score; the lowest 33% were classified as “Low”, and the highest 33% were classified as “High”. “Low” and “High” groups based on standardized *NUAK1* mRNA expression were generated in a similar manner, using *NUAK1* expression alone instead of a signature. These groups were then analyzed by Gene Set Variation Analysis^66^ with the option “Kernel estimation of the cumulative density function (kcdf)” set to “Poisson”, minimum gene set size set to 15, and using the MSigDB Hallmark Gene Set Collection (version 6.2)^45^.

### Statistical analysis

Statistical analyses were performed using GraphPad Prism 6.05 (GraphPad Software). Specific analysis details are described in figure legends.

## Supplementary Information

Supplementary Figures.

Supplementary Table S1.

Supplementary Table S2.

Supplementary Figures.
