# Supplementary material for: Loss of LKB1-NUAK1 signalling enhances NF-κB activity in a spheroid model of high-grade serous ovarian cancer
Source: Sci Rep. 2022 Feb 22;12:3011. doi: 10.1038/s41598-022-06796-2 (PMC8863794; doi:10.1038/s41598-022-06796-2)
Supplement: Supplementary file 1 — Supplementary Figures. [file 41598_2022_6796_MOESM1_ESM.pdf]

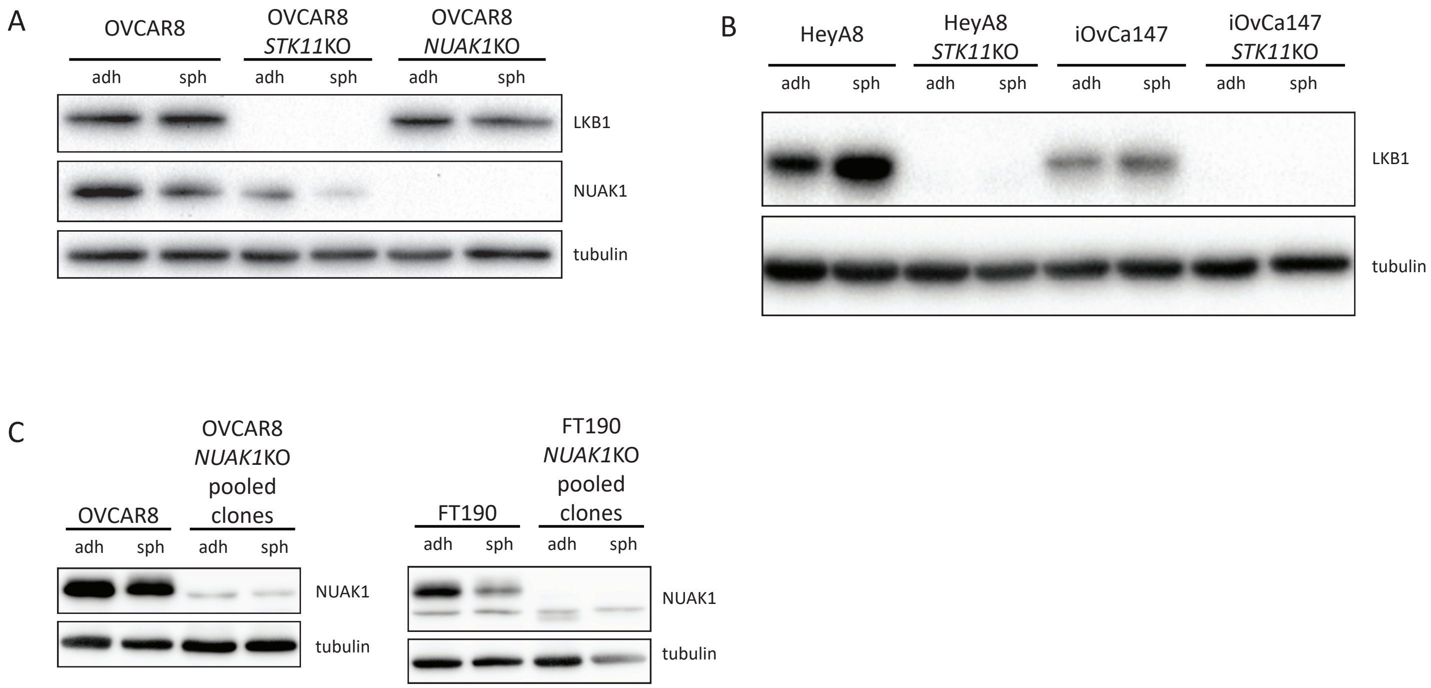

Supplementary Figure S1: Confirmation of LKB1 and NUAK1 loss in EOC and FT190 cell lines. Immunoblot analysis of LKB1 or NUAK1 in adherent (adh) and spheroid (sph) whole-cell lysates isolated from OVCAR8, OVCAR8-*STK11*KO, and OVCAR8-*NUAK1*KO EOC cells **(A)**, HeyA8, HeyA8-*STK11*KO, iOvCa147, and iOvCa147-*STK11*KO cells **(B)**, and OVCAR8-*NUAK1*KO and FT190-*NUAK1*KO populations of pooled clones **(C)**.

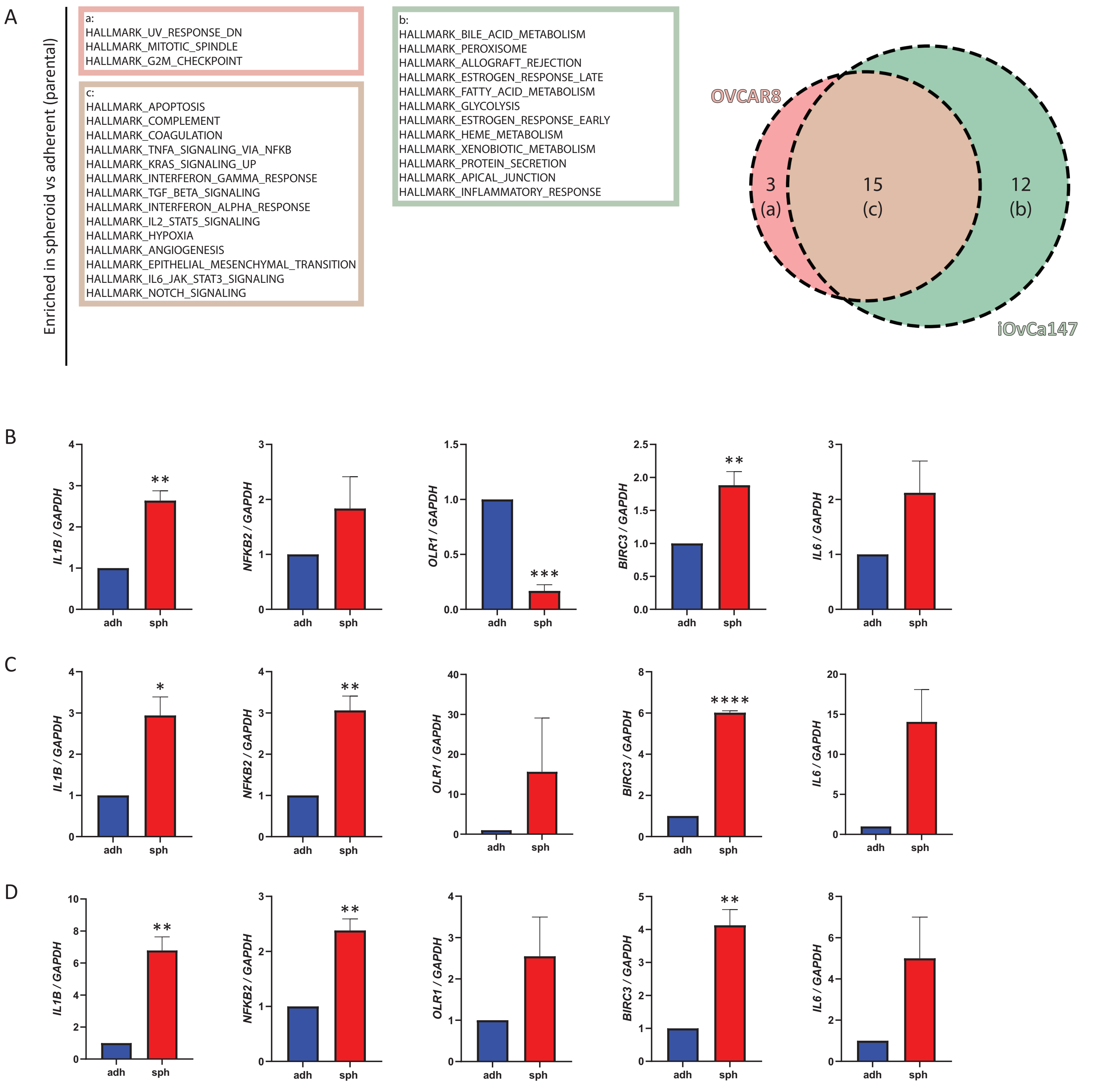

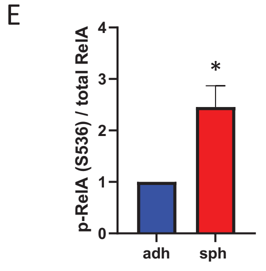

Supplementary Figure S2: Enhanced NF-κB signalling in EOC spheroids. (A) Transcriptional signatures enriched in OVCAR8 and iOvCa147 spheroid cells and compared with adherent cells, as determined by Gene Set Enrichment Analysis (BROAD Institute; p-value cutoff: 0.05, FDR cutoff: 0.25). **(B-D)** Expression of selected NF-κB target genes in adherent cells and spheroids using OVCAR8 **(B)**, iOvCa147 **(C)**, and HeyA8 **(D)** cell lines as measured by RT-qPCR. **(E)** Densitometric analysis of p-RelA (S536) in OVCAR8, iOvCa147, and HeyA8 cells grown in adherent and spheroid culture conditions. Data are shown as the mean for the three cell lines as spheroids normalized to adherent cells; representative immunoblots of these lysates are shown in Figure 2A.
Expression in spheroids were compared with adherent cells by unpaired, two-tailed Student's *t*-test (* $p \leq 0.05$, ** $p \leq 0.01$, **** $p \leq 0.0001$; $n = 3$). Error bars indicate standard error of the mean (S.E.M.).

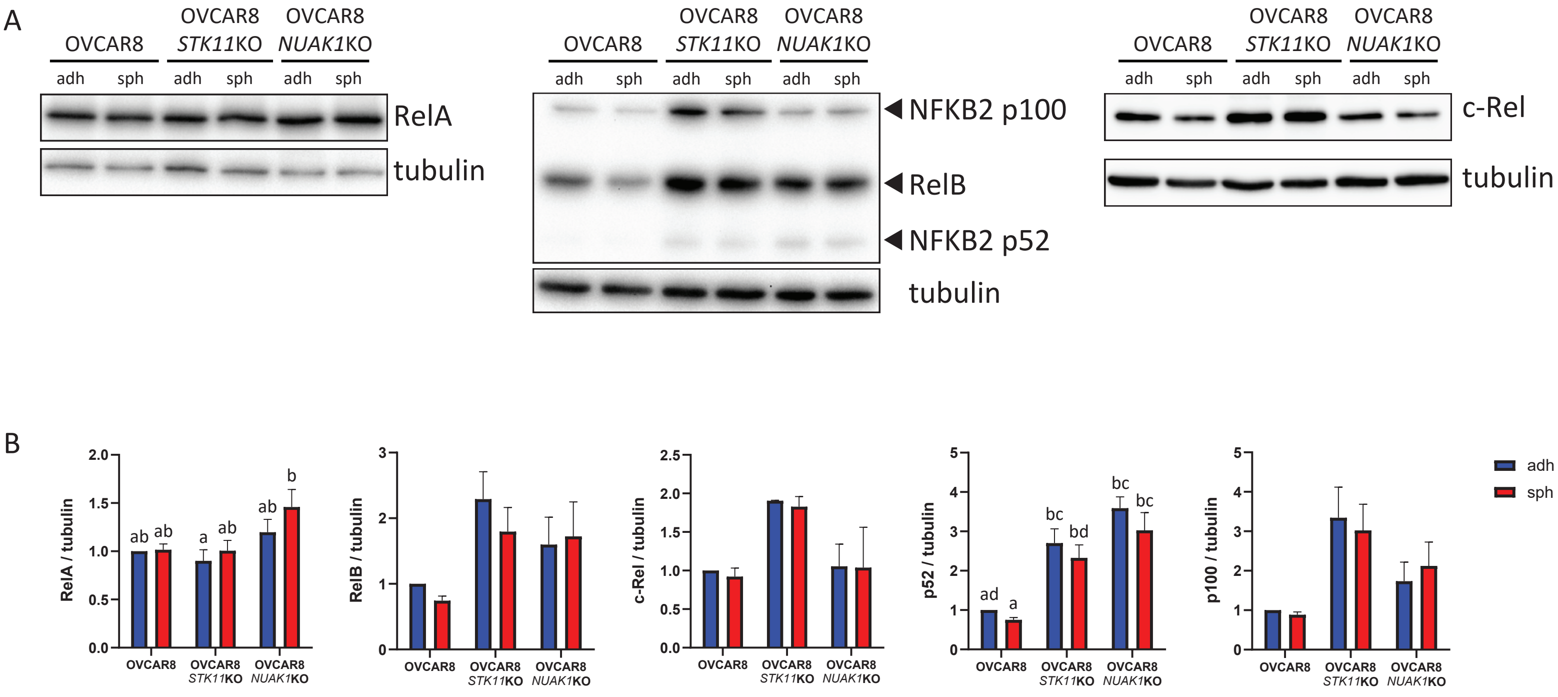

Supplementary Figure S3: Total abundance of NF-κB family transcription factors in OVCAR8-*STK11*KO and OVCAR8-*NUAK1*KO cells. (A) Representative immunoblots for NF-κB transcription factors in whole-cell lysates isolated from OVCAR8, OVCAR8-*STK11*KO and OVCAR8-*NUAK1*KO cells grown as adherent (adh) or spheroid (sph) cultures. **(B)** Densitometric analysis are shown as fold-change shown in blue (adherent culture) or red (spheroid culture) relative to adherent OVCAR8 cells. Statistical analysis was performed using two-way ANOVA and groups were compared by Tukey's multiple comparisons test with alpha set to 0.05, performing all pairwise comparisons; $n \geq 3$. Letter labels indicate statistically significant differences between groups. Error bars indicate standard error of the mean (S.E.M.).

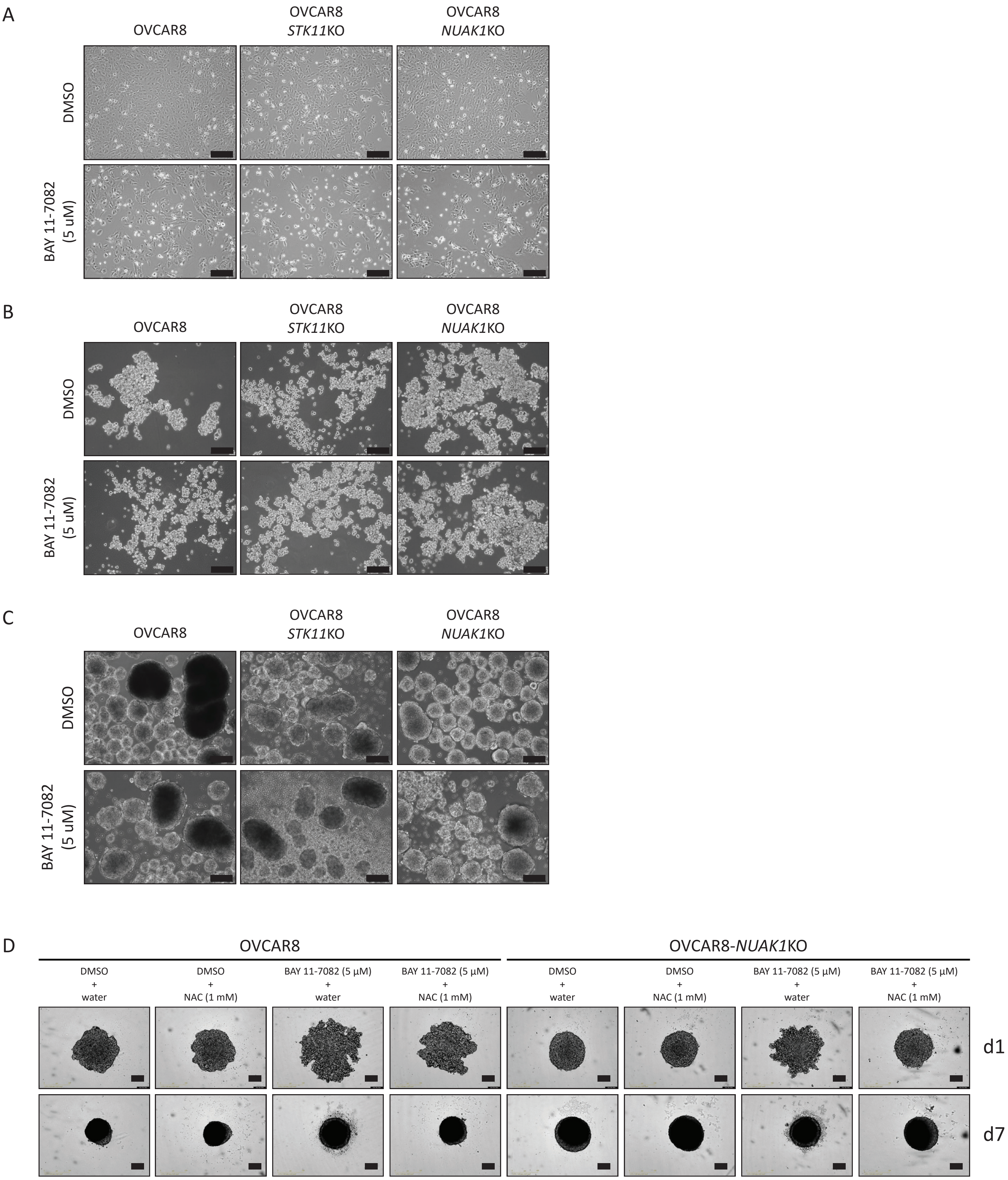

Supplementary Figure S4: Images of BAY 11-7082-treated EOC cells. Phase-contrast microscopy images of OVCAR8, OVCAR8-*STK11*KO, and OVCAR8-*NUAK1*KO cells treated with DMSO or 5 μM BAY 11-7082 for **(A)** two days in adherent culture, **(B)** one day in spheroid culture, or **(C)** seven days in spheroid culture. **(D)** Brightfield microscopy images of OVCAR8 and OVCAR8-*NUAK1*KO cells treated with DMSO or 5 μM BAY 11-7082 and water or 1 mM NAC for one (d1) and seven (d7) days in spheroid culture. Scale bars represent 200 μm.

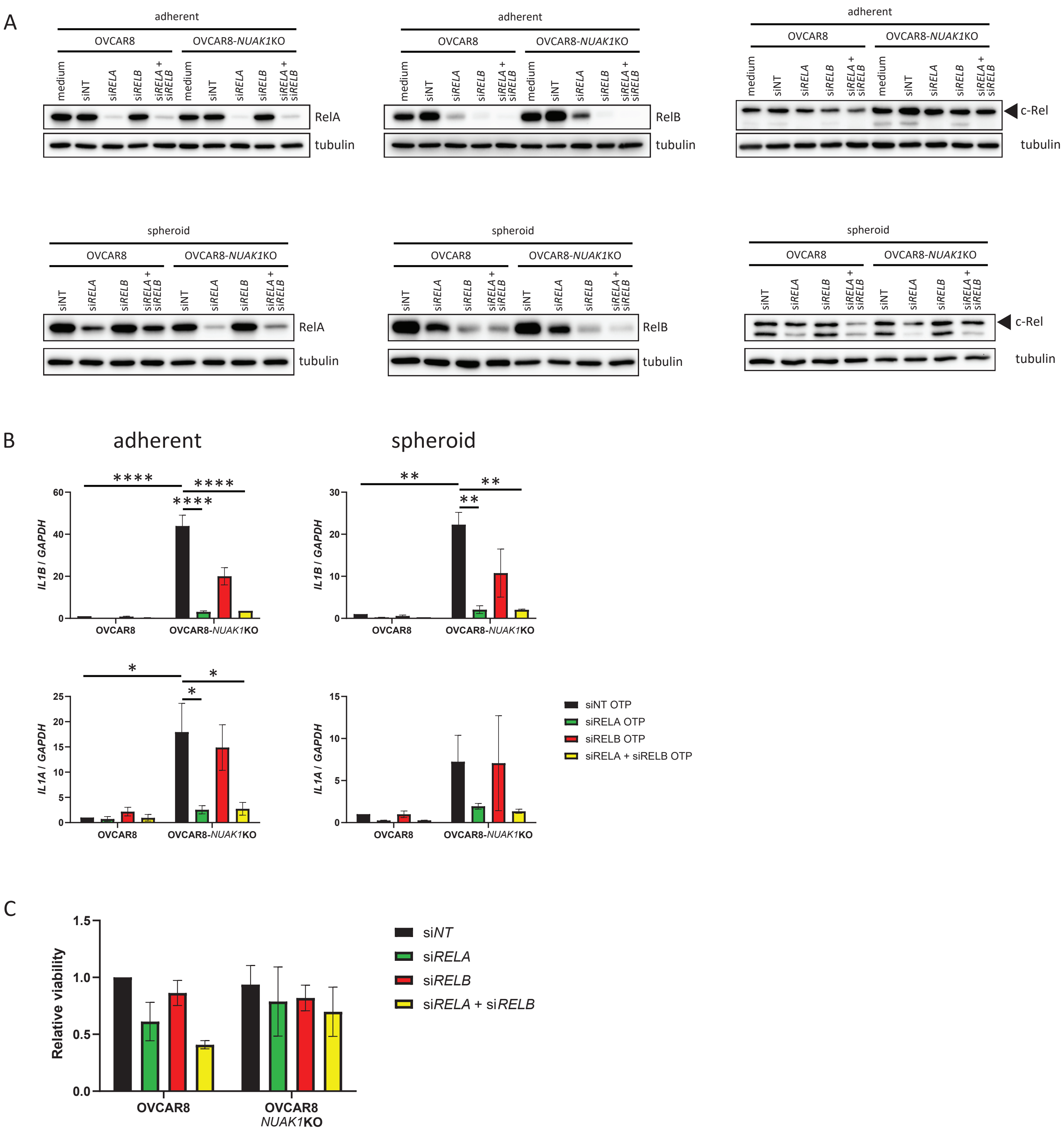

Supplementary Figure S5: Transient knockdown of RelA and RelB in EOC cells. **(A)** Representative immunoblots for RelA, RelB, and c-Rel in OVCAR8 and OVCAR8-*NUAK1*KO cells transfected with siNT (non-targeting control), si*RELA*, or si*RELB*, si*RELA*+si*RELB*, or untransfected (medium). Adherent (adh) cell lysates were collected three days post-transfection, and spheroid (sph) lysates were collected after seven days in spheroid culture. **(B)** Expression of NF-κB target genes, *IL1A* and *IL1B*, as determined by RT-qPCR. **(C)** Viability of OVCAR8 and OVCAR8-*NUAK1*KO cells after seven days in spheroid culture as measured by Trypan blue exclusion of dissociated spheroids. Statistical analysis was performed using two-way ANOVA and groups were compared by Tukey's multiple comparisons test with alpha set to 0.05, performing all pairwise comparisons; n = 2. For all bar graphs, error bars indicate standard error of the mean (S.E.M.).

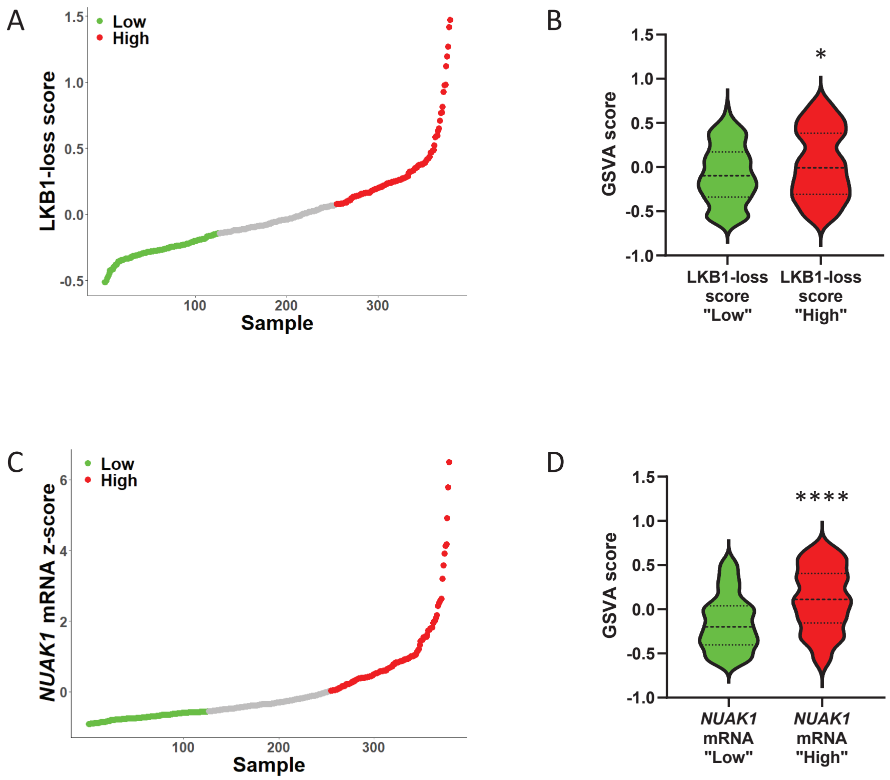

Supplementary Figure S6: HALLMARK_TNFA_SIGNALING_VIA_NFKB gene set variation analysis in ovarian tumours. (A) TCGA Ovarian Cancer samples ranked by LKB1-loss score, illustrating “Low” (bottom 33%) and “High” (top 33%) groups. **(B)** GSVA scores (HALLMARK_TNFA_SIGNALING_VIA_NFKB gene set) for sample groups shown in (A). **(C)** TCGA Ovarian Cancer samples ranked by NUAK1 mRNA z-score, illustrating “Low” and “High” groups. **(D)** GSVA scores (HALLMARK_TNFA_SIGNALING_VIA_NFKB gene set) for sample groups shown in (C). For (B) and (D), groups were compared by a two-tailed Mann-Whitney test (* $p \leq 0.05$, **** $p \leq 0.001$). Dashed line indicates the median for each group; dotted lines indicate quartiles.
