# Supplementary figures and images for: Loss of LKB1-NUAK1 signalling enhances NF-κB activity in a spheroid model of high-grade serous ovarian cancer
Source: Sci Rep. 2022 Feb 22;12:3011. doi: 10.1038/s41598-022-06796-2 (PMC8863794; doi:10.1038/s41598-022-06796-2)

Figure 2A

Figure 3A

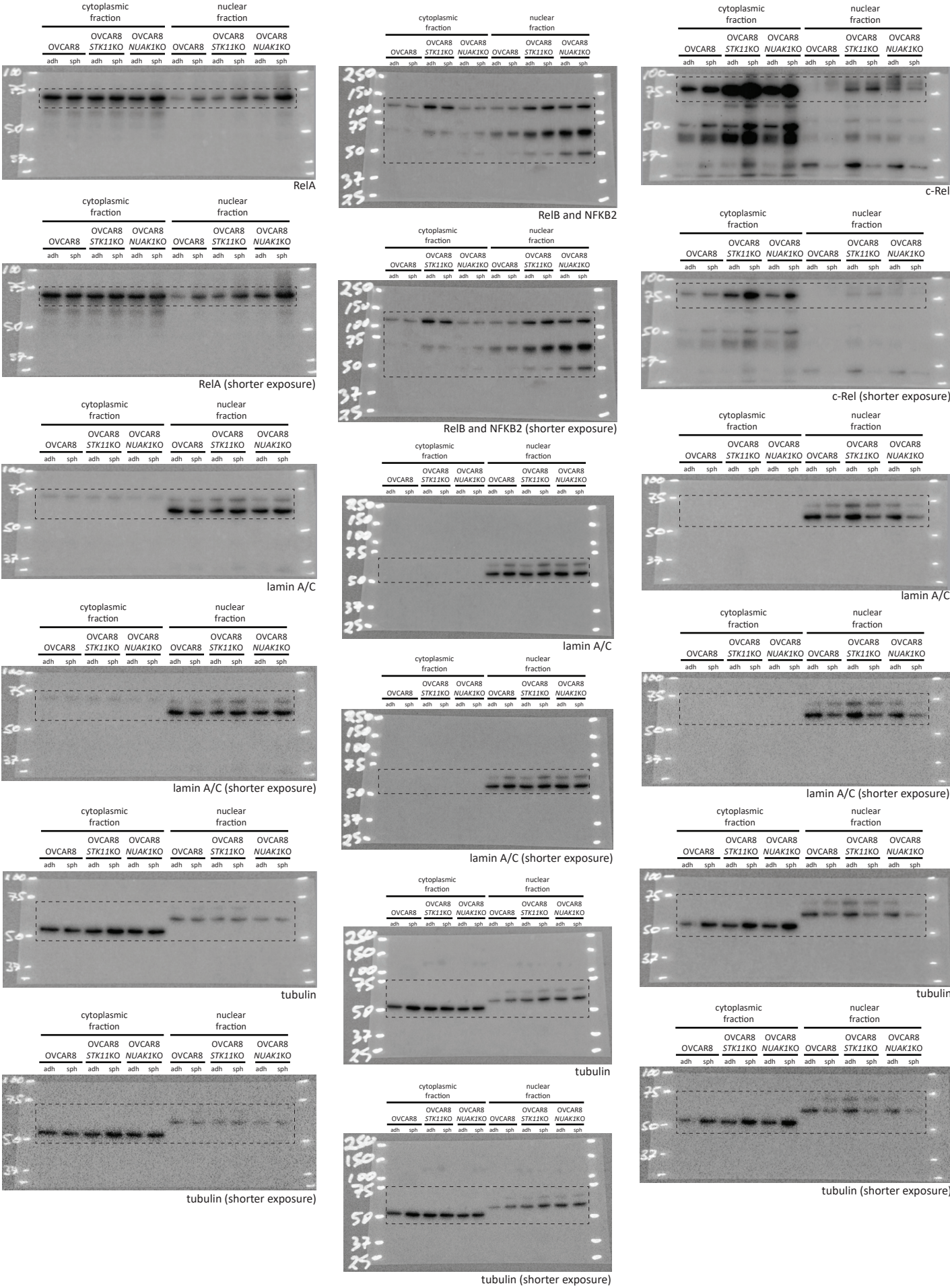

Figure 4A

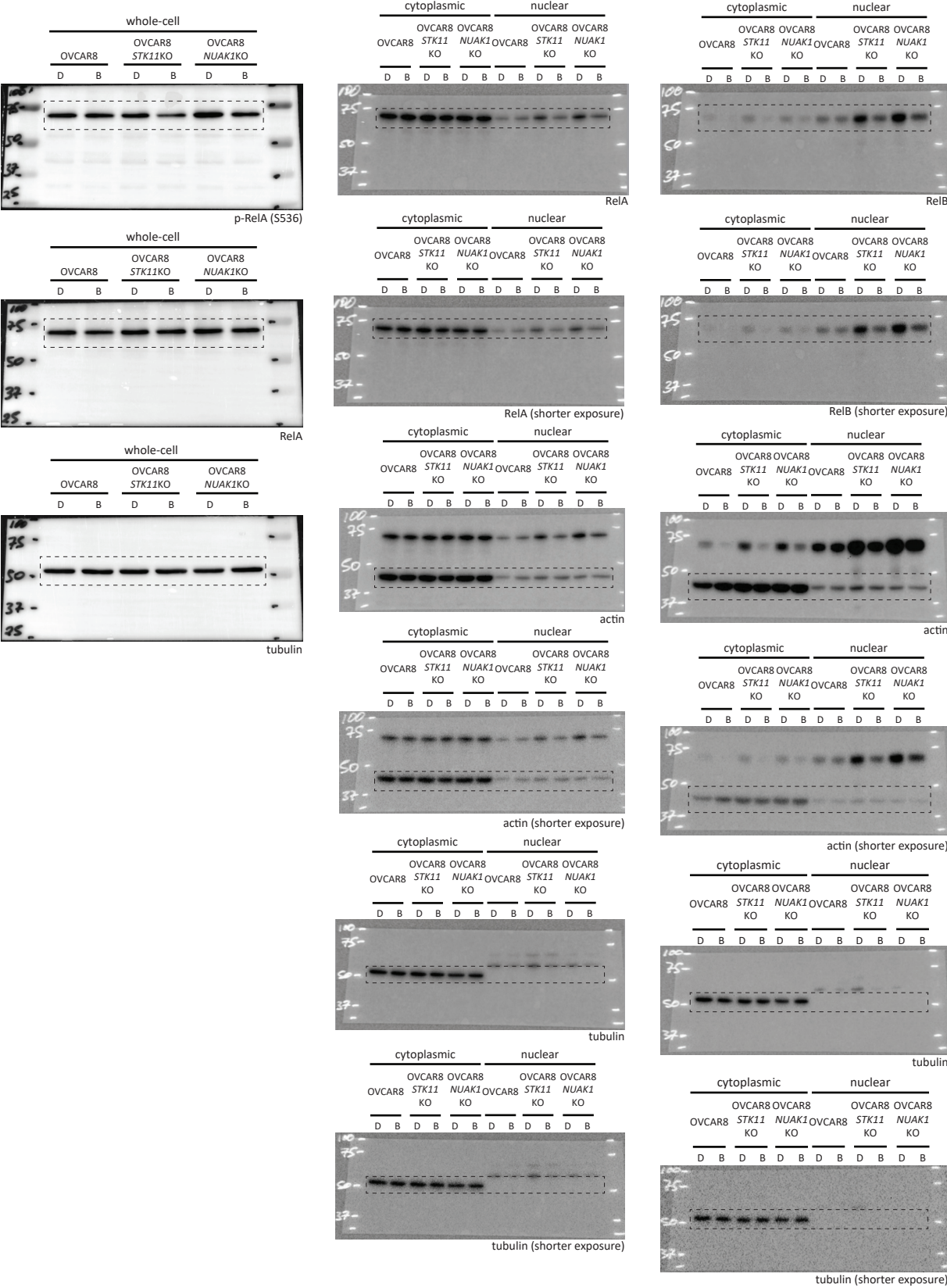

Figure 5A

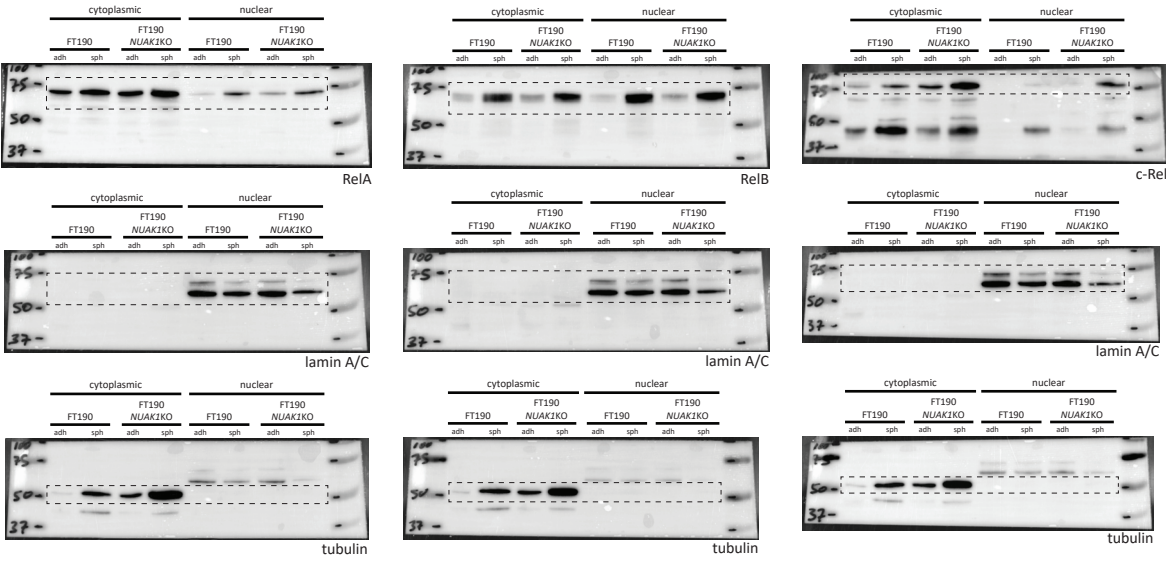

Figure S1

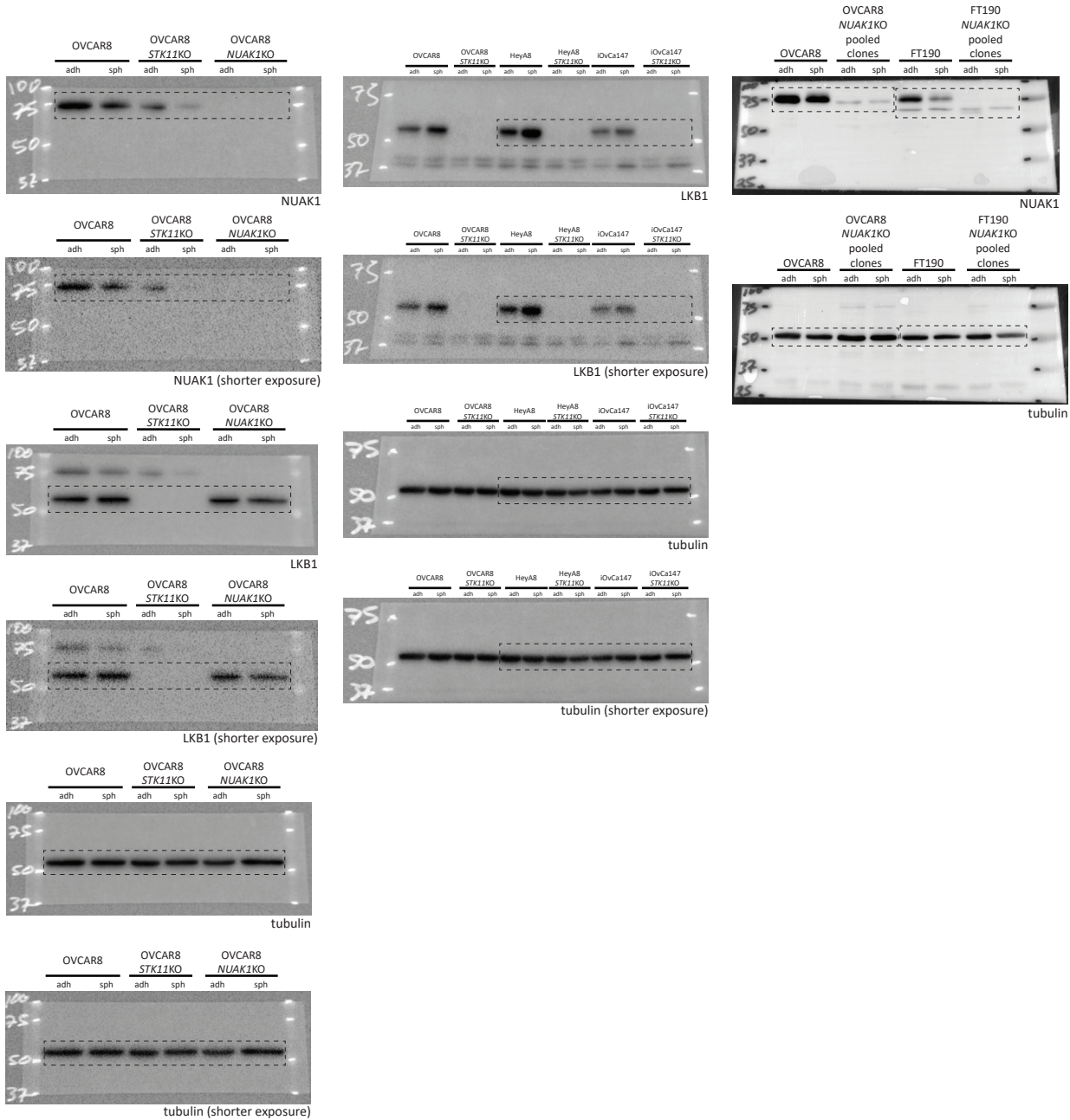

Figure S3A

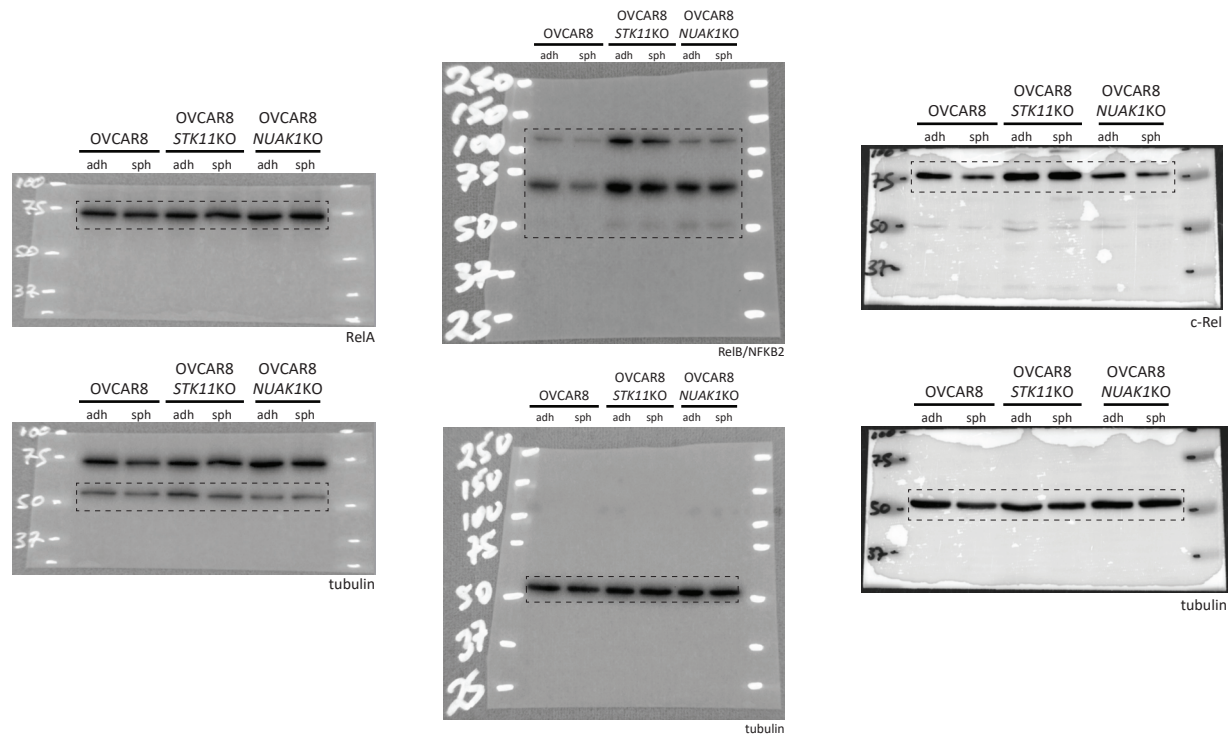

Figure S5A

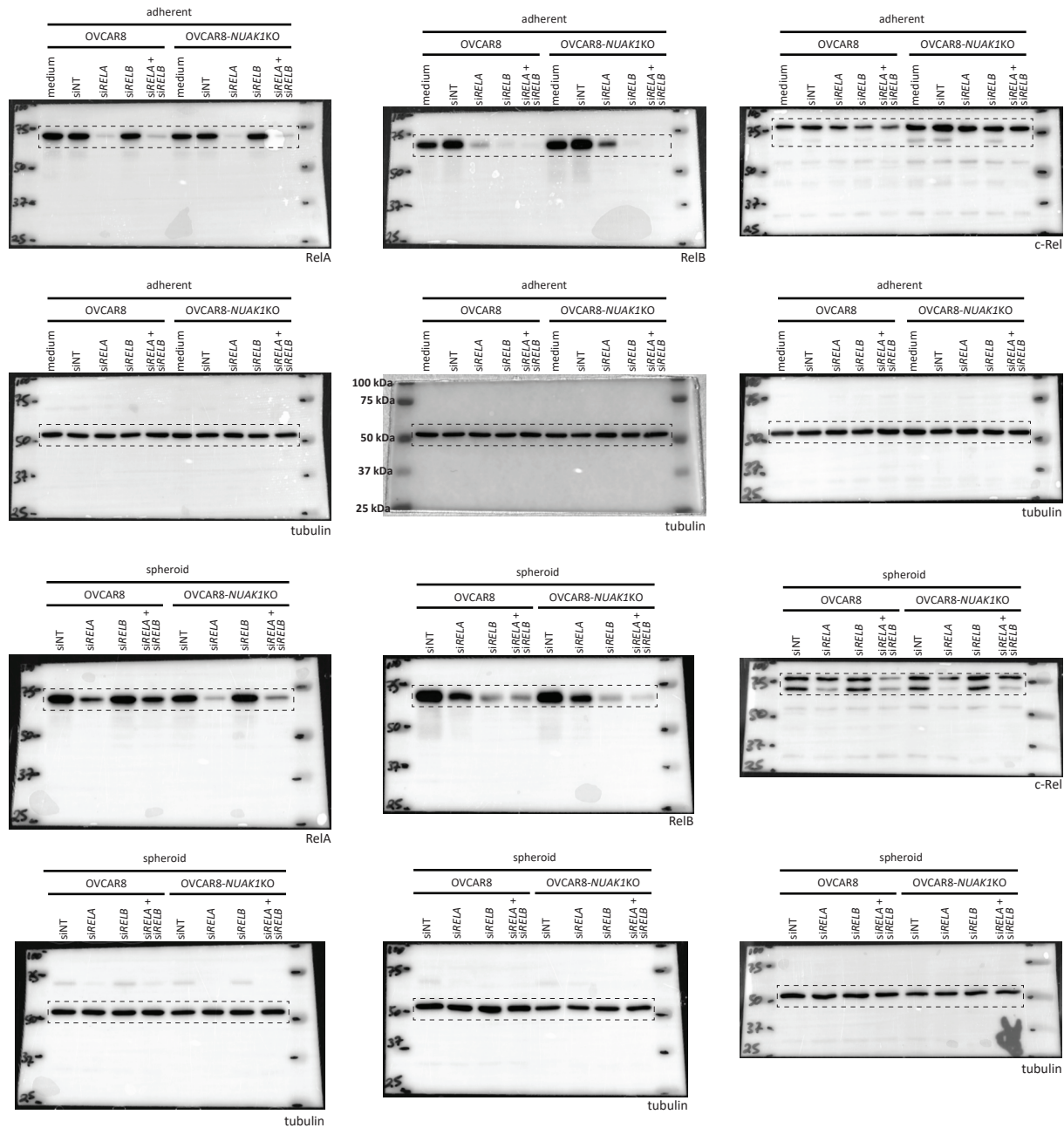

Supplement: Supplementary file 4 — Supplementary Figures. [file 41598_2022_6796_MOESM4_ESM.pdf]
